# Bandgap formation mechanism in tacticity inspired elastic mechanical metastructures

**DOI:** 10.1038/s41598-024-75462-6

**Published:** 2024-10-19

**Authors:** Ankur Dwivedi, Rajendra Kumar Munian, Bishakh Bhattacharya, Sondipon Adhikari

**Affiliations:** 1 Department of Mechanical Engineering, Indian Institute of Technology Kanpur, Kanpur, India; 2 Department of Mechanical Engineering, Indian Institute of Technology Ropar, Ropar, India; 3 James Watt School of Engineering, University of Glasgow, Glasgow, United Kingdom

**Keywords:** Mechanical metamaterials, Mechanical metastructures, Metabeam, Tacticity, Isotactic metabeam, Syndiotactic metabeam, Engineering, Mechanical engineering

## Abstract

Tacticity is long known as a significant contributor in changing the chemical and mechanical properties of the polymers drastically. This study explores mechanism of bandgap formation in elastic mechanical metastructures designed with a focus on tacticity. We introduce metabeams, comprising a primary slender beam embedded with short secondary beams featuring end masses at their tips. The investigation delves into the numerically simulated vibration characteristics of metabeams using finite element analysis, with a subsequent comparison to experimental results for fabricated metabeams. Employing a unit-cell design approach that manipulates spatial and physical parameters, we explore a wide range of uniform and non-uniform metabeam configurations based on the distance between secondary beams and distribution of local resonators as per tacticity. Hence, drawing inspiration from tacticity, we extend our investigation to isotactic and syndiotactic metabeams, altering physical parameters (mass) within the unit cell for both configurations. The strategic distribution of end masses on attached secondary beams introduces unique characteristics to isotactic and syndiotactic metabeams, allowing for the modulation of bandgaps without altering the natural frequencies of the resonators in symmetric and anti-symmetric metabeam designs. Our research demonstrates, incorporating tacticity in metabeam design offers a novel and unconventional approach to modulate the bandgap formation mechanism.

## Introduction

The research on the dynamics of periodic materials and structures has a profound historical background, from Newton’s first effort to study sound propagation in the air to Rayleigh’s investigation of continuous periodic structures. The periodic materials and structures demonstrate spatial periodicity, which can be in the geometry or constituent material phase or boundary conditions. This field of research has got another upsurge from the early 21st century and resulted in erudite studies on phononic crystals and metamaterials^1–3^. Metamaterials are artificially designed materials with repeated unit cells in their geometry to accomplish unusual physical properties; hence, they have been extensively investigated since the last decade^4–7^. Elastic mechanical metastructures are engineered materials with periodic or aperiodic repeated unit cells that can control elastic wave propagation^8,9^. Tailored geometry and pattern while designing metamaterials introduce unusual physical properties due to the added feature of local resonance and other attributes rarely observed in conventional materials^10,11^. These exceptional physical properties can give rise to idiosyncratic dynamic behavior such as negative stiffness, mass, and group velocity. These unusual dynamic properties have very little influence of the intrinsic properties of the constitutive material. On the other hand, phononic crystal is a composite material made up of multiple phases, which can be solid or fluid, periodically arranged in space to control acoustic or elastic properties. In phononic crystals, structural periodicity can be in geometry or the constitutive material phase and induces a high impedance difference, which primarily reinforces the formation of Bragg scattering attenuation bands. Bragg-type stopbands are formed at a higher frequency for a wavelength equal to the lattice size of the repeated unit cell due to destructive interference between propagated and reflected waves. However, if the lattice size is big enough, Bragg-scattering can be created in low-frequency also^12^. In the case of mechanical metamaterials, the attenuation band can be formed below the Bragg limit for wavelength much greater than the unit cell size^13^. Locally resonant units in the designed metastructure create subwavelength attenuation bandgap formation near the resonating frequency of the attached local resonators. The mass and stiffness of the resonator significantly affect the attenuation bandwidth. Moreover, the natural frequency of the resonator determines the frequency region of the attenuation band. Indeed, with the natural frequency, attenuation bandwidth increases; however, for the same natural frequency, the mass addition is the governing parameter. Hence, these physical properties of the local resonators can tune the attenuation bands at the subwavelength scale by manipulating elastic wave propagation in the designed metastructure^14,15^. On one side, vibration can be controlled in the primary structure, and on the other side, this energy can also be channelized for actively harvesting the dissipated vibrational energy^16,17^. It increases the functionality of mechanical metamaterials in a broad spectrum. The locally resonant units in mechanical metamaterials and metastructures facilitate the formation of an attenuation band near the resonant frequency by diverting the elastic wave energy from the primary structure^18^. Several numerical studies are reported in the literature to investigate flexural vibration bandgaps by considering Euler–Bernoulli or Timoshenko beam attached with discrete local resonators^19–21^. Discrete spring-mass resonators are suitable for understanding the bandgap characteristics on the frequency scale; however, implementation in actual field applications is impossible. Hence, researchers proposed beam-like resonators attached to the host beam. Inspired by these characteristics of metamaterials, Qureshi et al. proposed a 3D printed cantilever-in-mass structure having negative effective mass properties for creating stop bands in the desired frequency range^22^. Meng et al. have also investigated 3D-printed rainbow phononic crystals for vibration attenuation^23^. They proposed a unit cell with two cuboid blocks connected by curved beams to achieve a wider attenuation band. Earlier, Xiao et al. investigated locally resonant bandgap formation in the flexural beam attached with beam-like resonators^24^. They studied the interaction of locally resonant and Bragg bandgaps, near coupling effects in the proposed structure. Moreover, Wang et al. have also studied quasi-one-dimensional structure having periodically attached harmonic oscillators on slender beam^25^. They reported sub-frequency locally resonant highly asymmetric attenuation both theoretically and experimentally.

The recent active area of research in mechanical metamaterials includes studies on topological protected mechanical properties, which are independent of configurational change or deformations in the geometry^26–30^. Based on this, Huang et al. investigated zero-angle refraction of elastic waves in pseudospin-Hall phononic crystals^31^. They proposed an elastic near-zero refractive index metamaterial having a triangular lattice for achieving topological zero refraction. Further, they also proposed a ternary valley-Hall phononic crystals (PCs) with sub-wavelength topological negative refraction of elastic waves, which overcomes Bragg’s lattice-size restrictions^32^. While investigating captivating properties of mechanical metamaterials, Xia et al. studied topology in quasiperiodic resonant metastructure in which they reported additional bandgap apart from locally resonant bandgap^33^. The latest studies inspired from polymer science on tacticity in chiral phononic metamaterials suggest studying dispersion without changing stiffness and mass in the designed metastructure^34–36^. Tacticity is simply the way adjacent monomer units are arranged in a polymer. In polymer science, the orientation of adjacent monomer units leads to isotactic, syndiotactic, and atactic polymer designs^37^. Hitherto, studies on design and fabrication of tacticity-based elastic mechanical metamaterials have not been reported in the literature. Hence to fill this research lacuna, we investigate the bandgap formation mechanism in tacticity-based elastic mechanical metastructures through the vibration characteristics of the designed metabeams. These metabeams can be designed by varying the end mass distribution on the secondary beams, which alters dynamic properties of the system. The following section explains the originality of tacticity-based metabeams to perceive interesting dynamic properties.

## Results

**Fig. 1:**
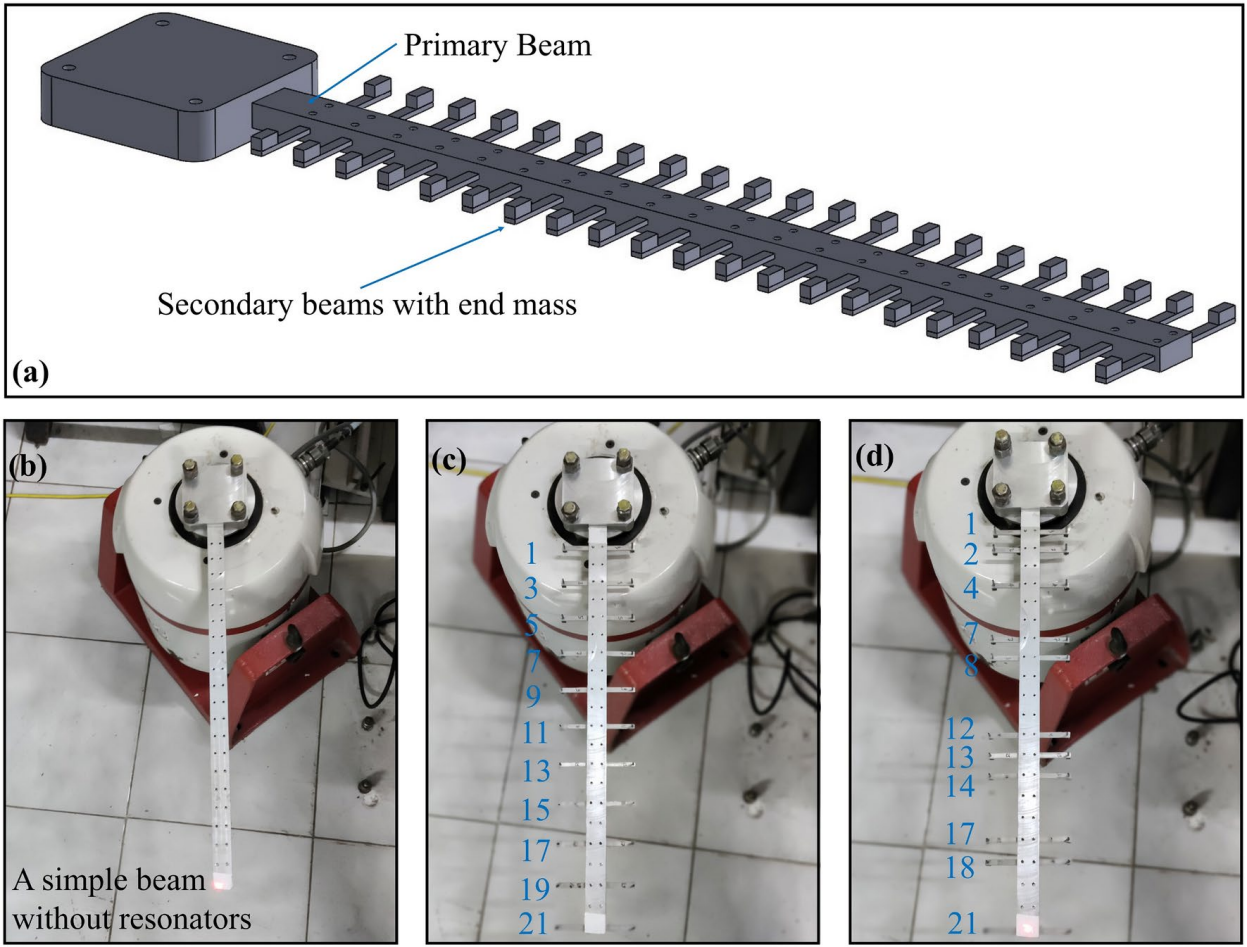
(**a**) A Beam based metastructure called metabeam is shown having continuous local resonators. In the metabeam, primary beam is embedded with secondary beams having end masss. The attached continuous secondary beams act like local resonators. (**b**) A simple beam without secondary beam attachments to the primary beam. The metabeam has 21 rectangular slots for attaching the secondary beams as local resonators. (**c**) Total 11 secondary beams are embedded to the primary beam starting from the root section at rectangular slot locations 1, 3, 5, 7, 9, 11, 13, 15, 17, 19 and 21 to form the uniform metabeam configuration in which the secondary beams are equally spaced from each other. (**d**) A non-uniform metabeam configuration in which 11 secondary beams can be attached at rectangular slot locations 1, 2, 4, 7, 8, 12, 13, 14, 17, 18, and 21.

Figure 1a portrays a conceptual illustration of the metabeam having primary and secondary beams. The primary beam is embedded with secondary beams having tip masses at both ends. These attached secondary beams act as a local resonator. The metabeam is designed and fabricated (see supplementary section 1.1) for comprehending the bandgap formation experimentally due to attached local resonators (secondary beam with end mass). The experimental results obtained for the fabricated metabeams are also well validated with finite element numerical models of metabeams in COMSOL Multiphysics. The wave propagation nature in the designed metastructure can be modulated by various unit-cell design techniques based on spatial parameter modification and configuration changes inspired by tacticity from polymer science. Following these design strategies, we propose uniform and non-uniform metabeams based on spatial parameter change. Figure 1b shows the primary beam having 21 rectangular slots uniformly spaced at 20 mm from their center point for attaching secondary beams. Figure 1c,d elucidate the actual locations of the secondary beams embedded in the primary beam to form uniform and non-uniform metabeams configurations. In the uniform metabeam, total 11 secondary beams having end masses *m* on both the tips are embedded in the primary beam at a fixed distance from each other. Whereas in the non-uniform metabeam, 11 secondary beams are embedded in the primary beam at varying distances from each other. For obtaining the non-uniform metabeam configurations the secondary beams must not be equally spaced. Hence, any combination of unequally spaced rectangular slots can be chosen as depicted in Fig. 1d. In mechanical metastructure, which has a finite dimension and a finite number of resonators, the optimal number of resonators required for bandgap appearance depends robustly on the targeted modal neighborhood (frequency range). More resonators are required to target higher modal neighbourhood existing in the high-frequency region. Moreover, an optimal number of resonators exists to target the broadest frequency bandgap, which is wider than the infinite resonator bandgap. The optimal number of resonators increases with a targeted modal neighborhood for a fixed mass ratio. Also, at a fixed targeted modal neighborhood (frequency), the optimal number of resonators increases with the mass ratio. Here, for monitoring the bandgap formation in the designed metabeam, 11 secondary beams are sufficient; however, more resonators are required to target higher vibrational modes. Uniform and non-uniform metabeams can be configured to observe the effect of increased end mass and number of secondary beams attached to the primary beam on the attenuation bandwidth (see supplementary section 1.4). Subsequently, by following the tacticity design technique of the unit cell from polymer science, we investigate isotactic and syndiotactic metabeams by varying the end mass distribution on the secondary beams for the uniform and non-uniform metabeam configurations. The bandgap formation is studied in all the above proposed metabeams configurations. Finally, the response of the secondary beam in the attenuation frequency region is also investigated to explore the energy harvesting possibilities from the designed metabeams.

There exists different mathematical techniques to estimate the bandgap in the mechanical metamaterials which can be based on the type of beam model assumption as per the unit-cell dimension^38^. The continuous resonators in Fig. 1a can be represented by the equivalent discrete resonators having mass (*m*) and stiffness (*k*) . Based on the geometrical information given about the designed metabeam (see supplementary section 1.1, Table S1 and Fig. S3), the unit cell of the metabeam will have slenderness ratio 2.0 ($L_u/t = 20/10 = 2.0$). Hence, based on slenderness ratio of the unit cell of metabeam, the analytical transfer matrix technique suggests formulating the equivalent metabeam using the Timoshenko beam theory. Subsequently, from the mathematical expression derived (see supplementary Equation S14), it can be concluded that the wave number is a function of the logarithmic of the eigenvalues of the transfer matrix. Therefore, the band structure of the metabeam can be plotted using this relation for a unit cell. However, the slenderness ratio defined by $L_u/t$ is not necessary to determine the choice of beam theory for dynamic modeling. Even for a thin beam with closely spaced resonators (having a small value of $L_u$), the ratio $L_u/t$ can be very large. Hence, the Euler–Bernoulli beam theory could still be sufficient for precise modeling. Additionally, if within the considered frequency range, the flexural wavelength is much longer than the thickness of the primary beam, there is no need to use the Timoshenko beam theory. The Euler–Bernoulli theory can make the modeling and analysis much more straightforward. Further, if we consider the Bragg scattering and local resonance interaction, the flexural wavelength of the primary beam at the resonance frequency of the local resonators (secondary beams) can be calculated. The Bragg scattering effects do not play an essential role if the spacing between adjacent resonators is much smaller than the flexural wavelength. However, if the spacing is comparable to the flexural wavelength, wave physics will become much more complex due to the interaction of local resonance and Bragg scattering effects^39^. Moreover, for the experimental investigation of bandgap, due to the effects of non-periodic constructions of the metabeam and boundary conditions of the finite structures, the experimentally demonstrated displacement transmissibility can not be well validated with conventional unit-cell based dispersion theory, which is developed for infinite periodic structures. Therefore, a comprehensive numerical analysis of the vibration characteristics of the metabeams by a well-established finite element software package can be done. The obtained numerical response of metabeams can be compared with the experimental results. However, perfect validation may not be possible as the experimental procedure involves unavoidable uncertainties in the fabrication, assembling with clamping hardware, and measurement of the specimens. In addition, while performing the experiments, additional modes like torsional may also be excited in the measurement. It may not be possible to consider unexpected uncertainties in the experiments, clamping hardware mass as an addition to end mass, including the potential inaccuracy and randomness of fabrications. However, considering structural damping about eigen frequency in the numerical model, we tried to obtain the best close match with frequency bandgaps and amplitudes. Considering all these factors, the experimental displacement transmittance response for every metabeam investigated is compared with the corresponding simulated metabeam response obtained numerically. Uniform and non-uniform metabeams and their isotactic and syndiotactic configurations due to the distribution of end masses on secondary beam attachments are analyzed numerically and experimentally. The numerical displacement transmittance is smooth and covers all the bandgaps obtained experimentally. The experimental displacement transmittance is not smooth due to unavoidable uncertainties and unexpected mode excitations during the experiment, as described earlier. The simulated displacement transmittance response shows bandgap formation in two or more frequency bandgaps due to the smoothness of the frequency function. In contrast, the frequency function is not smooth in experimental displacement transmittance response due to pseudo-random excitation. However, with the help of appropriate structural damping introduced in the system, all the frequency bandgap regions can be closely validated. For each metabeam investigated, constant structural damping between 1 and 2 $\%$ about the first two eigen frequencies of the metabeam is provided in the numerical model to match the bandgap and amplitude closely with experimental results. Following these guidelines, the numerical displacement transmissibility response of all the metabeams obtained using COMSOL Multiphysics is compared with the experimental results. The schematic for each metabeam is shown in respective panels with displacement transmittance response. It has details of secondary beam distribution about the mid-secondary beam on both sides of the metabeam and the magnitude of end masses at the tip. The displacement ratio of the endpoint to the base of the metabeam is shown on the y-axis to identify attenuation bands on the frequency spectrum. The excitation frequency spectrum up to 750 Hz through the frequency response function (FRF) is explored on the x-axis. The black curve shows the numerical transmissibility response of the metabeams, whereas the blue curve shows the experimental transmissibility response of metabeams. The reference line is shown in red, which helps in comprehending the displacement transmittance magnitude with respect to the zero line.

### Isotactic and syndiotactic metabeams

The secondary beam attachments to the primary beam facilitate the formation of locally resonant bandgaps in a frequency band. Hence, the elastic waves having a frequency close to eigen frequencies of the metabeam can be absorbed in these bandgaps (see supplementary Figs. S6a and S7a and corresponding discussion). The width of locally resonant bandgaps in a metastructure can be manipulated with physical properties of the attached resonators. The effect of increased end mass and the number of resonators on bandgap for a designed metabeam having uniform and non-uniform configurations can be investigated (see supplementary Figs. S6b,c,d and S7b,c,d). Increased end mass leads to shifting of attenuation band to the left on the frequency spectrum as the natural frequencies of attached secondary beams decreases. However, for targeting higher modes more number of resonators are required to get attenuation in higher frequency regions. Further inspired by polymer science, we are exploring the characteristics of resonant bandgaps using tacticity-based metabeam designs for uniform and non-uniform configurations. For experimental convenience, we keep the geometry unchanged and vary end masses on the secondary beams in all the metabeam configurations. We explore the uniform and non-uniform metabeam configurations based on isotactic, syndiotactic, and hybrid design combinations. These metabeams also have 11 secondary beams attached to the primary beam. About the mid-secondary beam in a metabeam, the end mass distribution on the secondary beams is changed to design isotactic, syndiotactic, or hybrid metabeam configurations. In every configuration, the mid-secondary beam is highlighted, and about it a particular pattern of secondary beams having end masses at the tip is followed to explore the locally resonant bandgap formation. The schematic of all metabeams investigated is shown along with displacement transmissibility in every panel having details of the secondary beam and end mass distribution about the mid-highlighted secondary beam. We categorize two subsections as per the type of secondary beams used in forming tacticity-based metabeam designs for the uniform and non-uniform configurations. In one subsection, the secondary beams have equal end masses at the tip, whereas in the following subsection, unequal end masses are present. The next two subsections explain the investigated tacticity-based metabeams for the uniform configuration.

#### Secondary beam having equal end masses in uniform metabeams

**Fig. 2:**
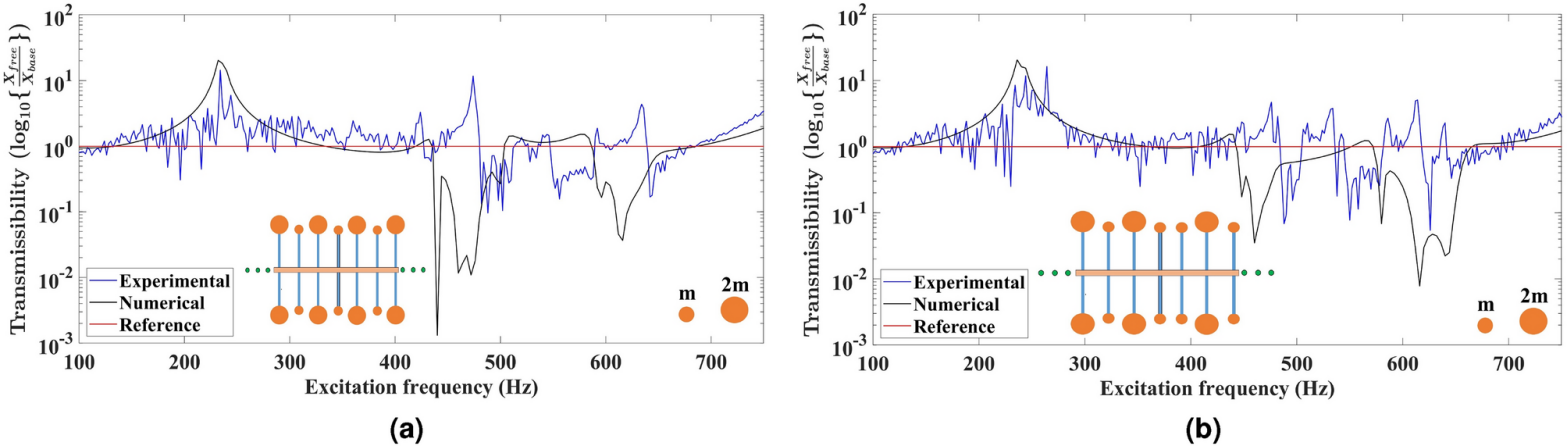
Comparison of the experimental and numerical displacement transmittance response for the uniform metabeams based on tacticity. The primary beam has **11** secondary beams embedded uniformly at a fixed distance from each other. The secondary beams at both tips have equal end masses to design the isotactic metabeams having uniform configurations as shown in (**a**) Isotactic symmetric array in which end mass distribution of ***2m–2m*** and ***m–m*** is followed about the mid highlighted secondary beam on both sides. (**b**) Isotactic anti-symmetric array in which end mass distribution of ***2m–2m*** and ***m–m*** on the secondary beams is followed on the first half of the metabeam and opposite mass distribution is present on the second half of metabeam.

In Fig. 2a, about the mid-highlighted secondary beam, the configuration is symmetric. The end mass distribution of ***2m–2m*** and ***m–m*** is followed about the mid-highlighted secondary beam to design an isotactic symmetric metabeam. The end masses having equal magnitude are placed on either side of the secondary beam. Hence, we have two types of secondary beams with different natural frequencies. The experimental displacement transmittance response shows the attenuation bandgaps in the frequency spectrum of 482–505 Hz, 552–592 Hz, and 640–680 Hz. There are two additional modes excited at frequencies 476 Hz and 638 Hz. On the other hand, the numerical displacement transmittance response shows bandgaps in frequency regions 452–502 Hz and 590–682 Hz. These two wider bandgaps are due to the two different natural frequencies of the attached secondary beams. In Fig. 2b, the configuration is anti-symmetric, which means about the mid highlighted secondary beam, the end mass distribution of ***2m–2m*** and ***m–m*** on the secondary beams is followed on the first half of the metabeam, and exactly opposite distribution of masses is present on the second half of metabeam. Hence, an isotactic anti-symmetric metabeam is formed with two types of resonators. The experimental displacement transmittance response shows the attenuation bandgaps in the frequency spectrum of 484–504 Hz, 544–582 Hz, 618–636 Hz, and 640–656 Hz. Due to other excited modes, there are peaks at 476 Hz, 614 Hz, and 640 Hz. Meanwhile, the numerical displacement transmittance response shows bandgaps in frequency regions 464–556 Hz and 580–656 Hz. Hence, due to the anti-symmetric distribution of secondary beams about the mid-secondary beam, both attenuation resonant bandgaps are swapped on the frequency spectrum. As the natural frequency of secondary beams is unchanged, the associated bandgap with lower amplitude moves left, and the other bandgap with high amplitude moves right. Due to negative group velocity the propagation of elastic waves in opposite direction occurs. Now the elastic waves with associated eigen frequency of the metabeam are absorbed in two swapped frequency bands.

#### Secondary beam having unequal end masses in uniform metabeams

**Fig. 3:**
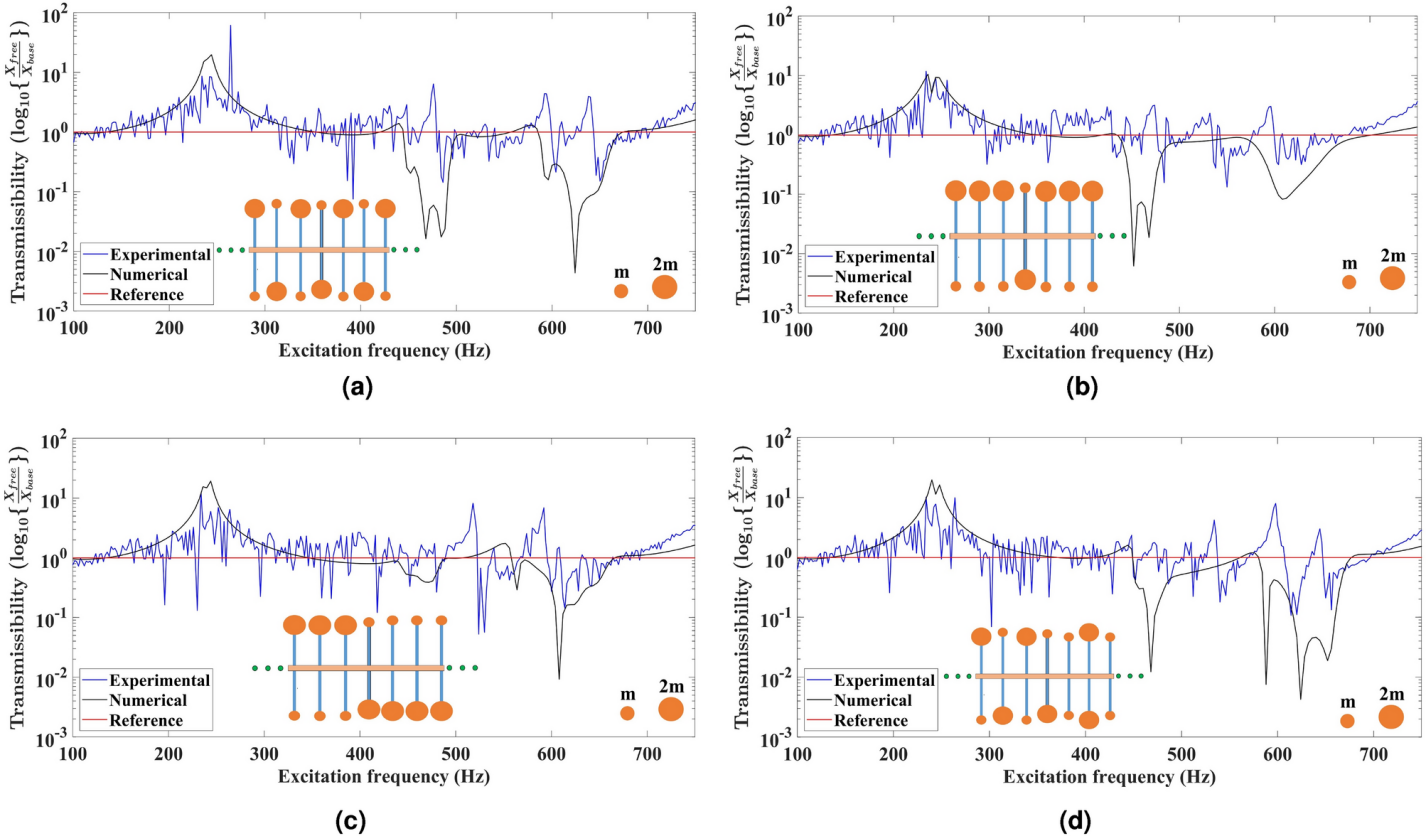
Comparison of the experimental and numerical displacement transmittance response for the uniform metabeam based on tacticity. The primary beam has **11** secondary beams embedded uniformly at a fixed distance from each other. The secondary beams at both tips have unequal end masses to design the metabeams based on tacticity having uniform configurations as shown in (**a**) Syndiotactic symmetric array in which end mass distribution of ***2m–m*** and ***m–2m*** is followed about the mid highlighted secondary beam. (**b**) Isotactic array in which end mass distribution of ***2m–m*** is shown on the entire beam. (**c**) Syndiotactic array in which end mass distribution of ***2m–m*** on the first half and ***m–2m*** on another half about the highlighted secondary beam. (**d**) Syndiotactic array in which end mass distribution of ***2m–m*** and ***m–2m*** is shown on the first half of the metabeam about the highlighted secondary beam. The other half of the metabeam is isotactic and has an end mass distribution of ***m–m*** and ***2m–2m***.

Figure 3a,b,c show the configurations of metabeams in which end masses on all the secondary beams are unequal in magnitude. In Fig. 3a, the end mass distribution of ***2m–m*** and ***m–2m*** is followed about the mid highlighted secondary beam to design a syndiotactic symmetric metabeam. The experimental displacement transmittance response shows the attenuation bandgaps in the frequency spectrum of 456–464 Hz, 480–488 Hz, and 644–668 Hz. The additional excited mode has a peak at 476 Hz. The numerical displacement transmittance response shows bandgaps in frequency regions 456–488 Hz and 594–670 Hz. The configuration shown in Fig. 3b has an end mass distribution of ***2m–m*** on either side of the highlighted secondary beam to design an isotactic metabeam. The experimental displacement transmittance response shows the attenuation bandgaps in the frequency spectrum of 428–444 Hz, 448–460 Hz, 476–484 Hz, 490–512 Hz, 532–576 Hz, and 600–668 Hz. The additional two excited modes have peaks at 472 Hz and 596 Hz, resulting in the fragmentation of resonant bandgaps. On the other side, the numerical displacement transmittance response shows a bandgap in frequency region 452–693 Hz; however, the amplitude of the bandgap is low in frequency regions 492–584 Hz and 648–693 Hz. Figure 3c has end mass distribution of ***2m–m*** on one side and ***m–2m*** on another side of the highlighted secondary beam to design a syndiotactic metabeam. The experimental displacement transmittance response shows the attenuation bandgaps in the frequency spectrum of 416–424 Hz, 450–460 Hz, 478–488 Hz, 524–568 Hz, and 596–672 Hz. Due to different mode excitations, there are peaks at frequencies 478 Hz and 644 Hz. The numerical displacement transmittance response shows bandgaps in frequency regions 444–480 Hz and 584–670 Hz. If we compare the displacement transmittance response from the previous configuration in Fig. 3b, due to exactly opposite distribution of end mass ***m–2m*** on the right half of the metabeam, the local resonant bandgaps are swapped. The bandgap with lower amplitude moves left, and the other with high amplitude moves right. It is due to the propagation of elastic waves in opposite direction having negative group velocity. However, the natural frequency of resonators is unchanged. The other syndiotactic metabeam configuration as shown in Fig. 3d is hybrid case in which equal and unequal end masses have been attached on either side of the secondary beam. It has end mass distribution of ***2m–m*** and ***m–2m*** on the left side of the highlighted secondary beam. The right side has ***m–m*** and ***2m–2m*** end mass distribution on secondary beams. The experimental displacement transmittance response shows the attenuation bandgaps in the frequency spectrum of 464–508 Hz, 540–570 Hz, 608–632 Hz, and 648–688 Hz. There are peaks at frequencies 476 Hz, 536 Hz, 600 Hz, and 646 Hz in between the bandgaps due to other mode excitations. The numerical displacement transmittance response shows bandgaps in frequency regions 464–556 Hz and 574–679 Hz. Here, we can see three resonant bandgaps due to three secondary beams acting as resonators having different natural frequencies. The other possible hybrid metabeam (see supplementary Fig. S8a) can be also designed to have only two resonant bandgaps by two types of secondary beams.

Likewise, the previously investigated isotactic and syndiotactic metabeam designs for the uniform configuration, the same type of metabeams are also explored for the non-uniform configuration. Now, the secondary beams are embedded in the primary beam at varying distances from each other, and the arrangement of end masses at the tip is changed about the highlighted mid-secondary beam to form isotactic and syndiotactic metabeams. The below two subsections explain all the investigated tacticity-based metabeams for the non-uniform configuration.

#### Secondary beam having equal end masses in non-uniform metabeams

**Fig. 4:**
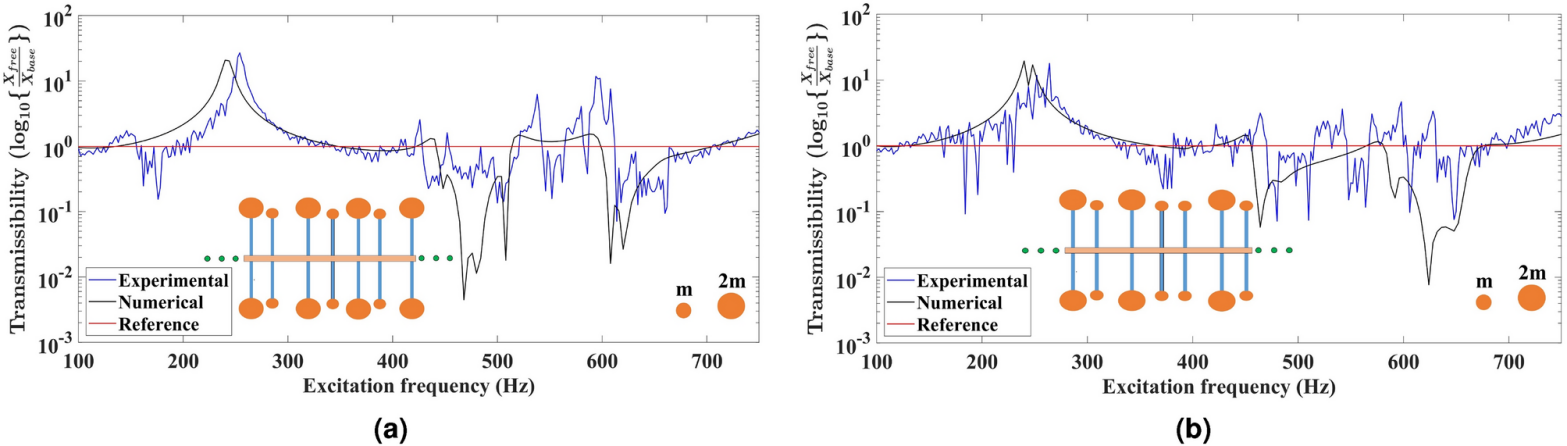
Comparison of the experimental and numerical displacement transmittance response for the non-uniform metabeam based on tacticity. The primary beam has **11** secondary beams embedded non-uniformly at varying distances from each other. The secondary beams at both tips have equal end masses to design the isotactic metabeams having non-uniform configurations as shown in (**a**) Isotactic symmetric array in which end mass distribution of ***2m–2m*** and ***m–m*** is followed about the mid highlighted secondary beam on both sides. (**b**) Isotactic anti-symmetric array in which end mass distribution of ***2m–2m*** and ***m–m*** on the secondary beams is followed on the first half of the metabeam and opposite mass distribution is present on the second half of metabeam.

Figure 4a is having end mass distribution of ***2m–2m*** and ***m–m*** on both sides of the highlighted secondary beam to design an isotactic symmetric metabeam. Hence, we have two types of resonators with different natural frequencies. The experimental displacement transmittance response shows the attenuation bandgaps in the frequency spectrum of 428–450 Hz, 456–520 Hz, and 612–705 Hz. There are peaks at frequencies 452 Hz and 594 Hz in bandgaps due to other excited modes. The numerical displacement transmittance response shows bandgaps in frequency regions 440–516 Hz and 600–704 Hz. The configuration shown in Fig. 4b is anti-symmetric, which means the secondary beams have end mass distribution of ***2m–2m*** and ***m–m*** on the left side of highlighted mid secondary beam. In contrast, the right side has ***m–m*** and ***2m–2m*** end mass distribution on secondary beams. The experimental displacement transmittance response shows the attenuation bandgaps in the frequency spectrum of 468–516 Hz, 552–573 Hz, 598–608 Hz, and 628–684 Hz. There are peaks at frequencies 466 Hz, 592 Hz, and 622 Hz in between the bandgaps due to the excitation of additional modes. The numerical displacement transmittance response shows bandgaps in frequency regions 464–572 Hz and 594–683 Hz. By including tacticity-based metabeam designs with equal end masses on secondary beams in the non-uniform configuration, we observe wider attenuation bands than uniform metabeams.

#### Secondary beam having unequal end masses in non-uniform metabeams

**Fig. 5:**
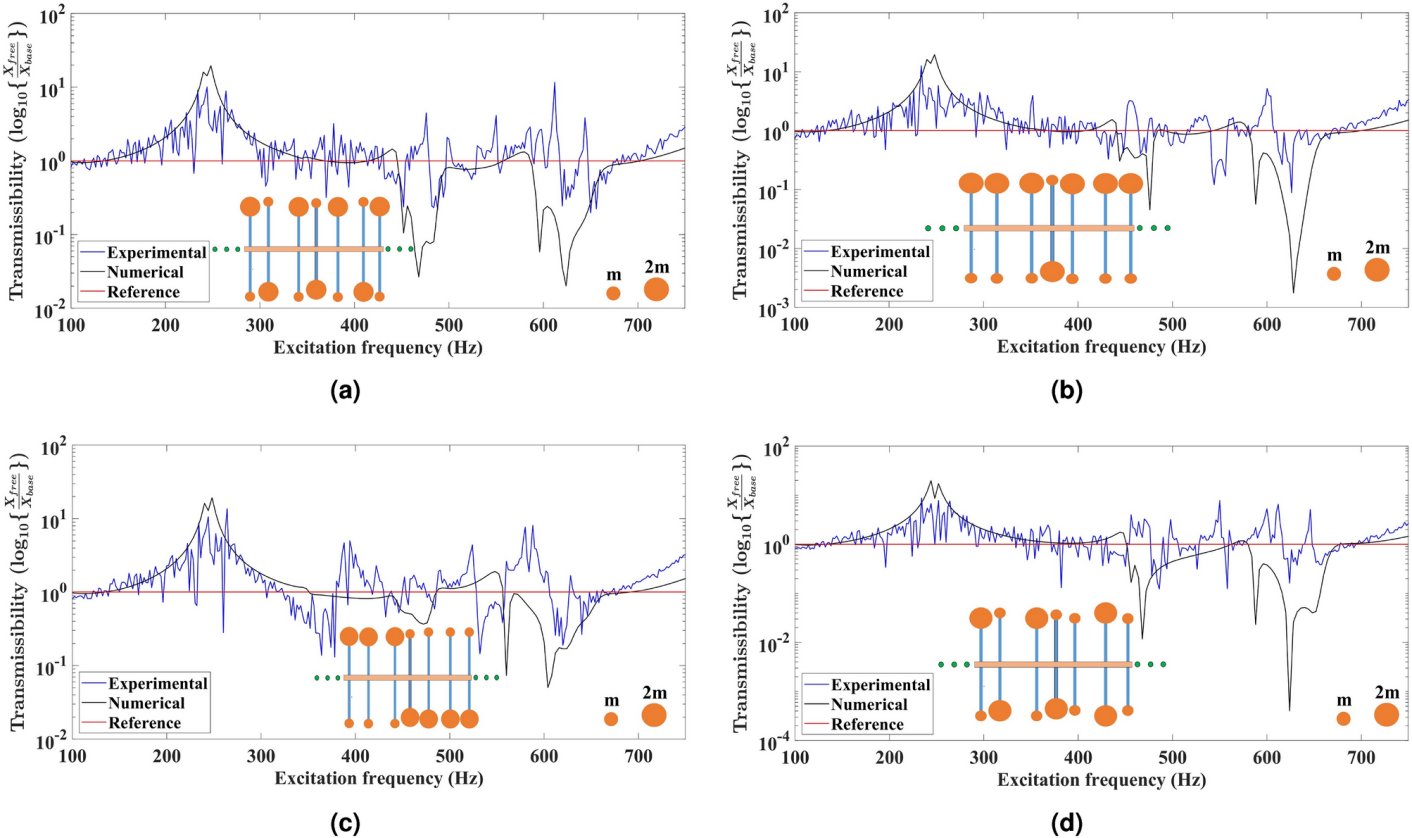
Comparison of the experimental and numerical displacement transmittance response for the non-uniform metabeam based on tacticity. The primary beam has **11** secondary beams embedded non-uniformly at varying distances from each other. The secondary beams at both tips have unequal end masses to design the metabeams based on tacticity having non-uniform configurations as shown in (**a**) Syndiotactic symmetric array in which end mass distribution of ***2m–m*** and ***m–2m*** is followed about the mid highlighted secondary beam. (**b**) Isotactic array in which end mass distribution of ***2m–m*** is present on the entire beam. (**c**) Syndiotactic array in which end mass distribution of ***2m–m*** on the first half and ***m–2m*** on another half about the highlighted secondary beam. (**d**) Syndiotactic array in which end mass distribution of ***2m–m*** and ***m–2m*** on the first half of the metabeam about the highlighted secondary beam. Another half of the metabeam is isotactic and has an end mass distribution of ***m–m*** and ***2m–2m***.

The configurations shown in figures Fig. 5a,b,c have unequal end masses at both tips of the secondary beam. In all configurations, the highlighted secondary beam at the middle has end mass ***m-2m*** at both tips. Figure 5a has end mass distribution of ***2m–m*** and ***m–2m*** on both sides of the highlighted mid-secondary beam for designing. The experimental displacement transmittance response shows the attenuation bandgaps in the frequency spectrum of 420–456 Hz, 476–496 Hz, 504–528 Hz, 552–560 Hz, 600–606 Hz, 616–640 Hz, and 644–684 Hz. There are peaks at frequencies 475 Hz, 642 Hz, and 612 Hz in bandgaps due to other excited modes. The numerical displacement transmittance response shows bandgaps in frequency regions 442–556 Hz and 594–704 Hz. The configuration shown in Fig. 5b is having ***2m–m*** end mass distribution on both sides of the highlighted secondary beam to design an isotactic metabeam. The experimental displacement transmittance response shows the attenuation bandgaps in the frequency spectrum of 420–452 Hz, 464–476 Hz, 496–516 Hz, 540–560 Hz, and 608–668 Hz. Due to additional excited modes, there are peaks at frequencies 454 Hz and 600 Hz. The numerical displacement transmittance response shows bandgaps in frequency regions 452–494 Hz, 496–540 Hz, and 594–692 Hz. In Fig. 5c about the mid highlighted secondary beam on the right side end mass distribution of ***2m–m*** is present on secondary beams and on left side ***m–2m*** end mass distribution is shown to design syndiotactic symmetric metabeam. The experimental displacement transmittance response shows the attenuation bandgaps in the frequency spectrum of 312–372 Hz, 428–452 Hz, 460–484 Hz, 524–552 Hz, 600–626 Hz, and 632–672 Hz. There are peaks at frequencies 384 Hz and 578 Hz in bandgaps due to other excited modes present. The numerical displacement transmittance response shows bandgaps in frequency regions 352–480 Hz and 550–678 Hz. Here, we observe no swapping in locally resonant bandgaps by changing the opposite distribution of end masses on secondary beams around the mid resonator in contrast to uniform metabeam configuration. The syndiotactic metabeam configurations Fig. 5d is the hybrid case in which one part of the metabeam has unequal end masses and another part has equal end masses on both tips of the secondary beam. The attached secondary beams have end mass distribution of ***2m–m*** and ***m–2m*** on the left side about the highlighted mid secondary beam. On the right side, the secondary beams have ***m–m*** and ***2m–2m*** end mass distribution to design a syndiotactic metabeam. The experimental displacement transmittance response shows the attenuation bandgaps in the frequency spectrum of 424–448 Hz, 472–494 Hz, 508–528 Hz, 552–564 Hz, 616–640 Hz and 646–684 Hz. The bandgaps have peaks at frequencies 496 Hz, 548 Hz, 610 Hz, and 642 Hz. The numerical transmittance response shows bandgaps in frequency regions 456–564 Hz and 582–684 Hz. Further, another hybrid metabeam configuration (see supplementary Fig. S8b) has been also investigated. We observe that inclusion tacticity results in a broader attenuation bandwidth than uniform metabeam in the non-uniform configuration.

### Response of the secondary beam for energy harvesting prospect

**Fig. 6:**
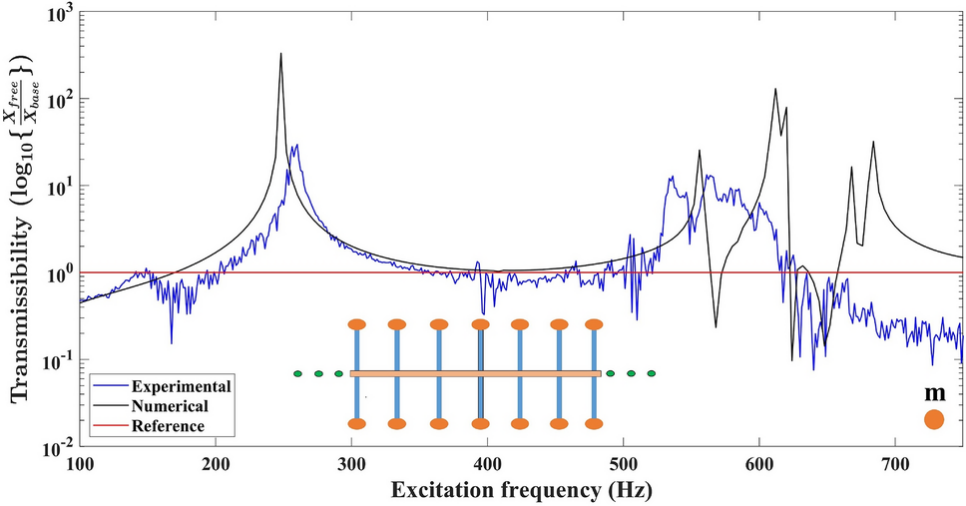
Comparison of the experimental and numerical displacement transmittance response for the secondary beam. In a uniform metabeam embedded with **11** secondary beams acting as local resonators having tip mass **m**, the response of the secondary beam attached at location **6** is measured.

Hitherto, we have seen the secondary beams acting as resonators facilitate bandgap formation near their natural frequencies. It implies that the elastic waves cannot propagate along the primary beam in the bandgap frequency region. Hence, the attached secondary beams vibrate with high amplitude. Therefore, the inclusion of energy-harvesting material in the secondary beams can be advantageous for simultaneous vibration attenuation and energy harvesting. Here, in a metabeam, the response of the secondary beam is studied to check the possibility of energy harvesting. Figure 6 exhibits the response of the secondary beam in a uniform metabeam. Here, we measure the displacement transmittance response of the secondary beam at location **6**, just after the mid-highlighted secondary beam. The displacement transmittance is obtained by dividing the secondary beam’s tip displacement by the primary beam’s base displacement. When the transmissibility response of the secondary beam at the sixth location in metabeam is observed, it can be easily perceived in the frequency region 520–616 Hz; the amplitude of tip displacement is significant, with peaks at 536 Hz and 563 Hz. The displacement transmissibility plot for the secondary beam at the sixth location is also simulated numerically for the uniform metabeam represented. The tip displacement is maximum in the frequency region 528–620 Hz with peaks at 565 Hz, 612 Hz, and 684 Hz. For the uniform metabeam explained in the supplementary Fig. S6a, the experimental bandgaps were observed in 544–572 Hz and 616–642 Hz. It implies the elastic energy flows to secondary beams acting as resonators in the attenuation frequency range. Hence, the displacement transmissibility response shows the secondary beam displacement is out of phase with the primary beam. If energy-harvesting material like a piezoelectric is embedded in the secondary beam, energy can be harvested due to the deformation of the piezoelectric crystal.

## Discussion

To summarize everything, we have shown the realistic metastructure architecture, insights into disorder effects, novel metabeam designs based on tacticity, rigorous experimental validations, and exploration of dual functionalities that offer essential advancements to the field of locally resonant metamaterials. The work provides both fundamental insights and practical guidance for engineering applications. Table 1 summarizes the important observations concluded while investigating metabeam designs for the uniform and non-uniform configurations.

**Table 1:** Bandgap characteristics of the metabeams.

Uniform/non-uniform metabeams	
Effect of end mass	Effect of resonators
Bandgap shifts to lower frequency region by increasing end mass on secondary beams due to decreased natural frequency.	An increased number of secondary beams in metabeam shifts the bandgap in the higher frequency region, hence bandgaps in higher frequency region can be achieved.

Tacticity in uniform metabeams	
Equal end mass distribution	Unequal end mass distribution
Two frequency bandgaps are observed on the frequency spectrum. Symmetric and anti-symmetric isotactic metabeam designs can swap these bandgaps without changing the natural frequencies of the resonators.	Two frequency bands are also observed for syndiotactic metabeams. Isotactic and syndiotactic metabeam designs can swap these bandgaps. In hybrid cases, two or more frequency bands can be seen.

Tacticity in non-uniform metabeams	
Equal end mass distribution	Unequal end mass distribution
Two wide frequency bands are observed in isotactic symmetric and anti-symmetric metabeam designs compared to uniform metabeam configurations.	Wider frequency bands are observed for all the configurations, including hybrid cases. However, bandgaps can not be swapped by including tacticity.

This paper introduces a transformative approach to metastructure design, emphasizing the practicality of continuous metabeams with embedded beam resonators. Departing from theoretical ideals, our work establishes a tangible foundation for locally resonant metamaterials, marking a paradigm shift in how we conceive and engineer such structures. The incorporation of tacticity, inspired by polymer science, allows for innovative mass distribution concepts that provide fabrication-friendly means to finely modulate bandgap characteristics in metastructures. Our investigation into uniform and non-uniform resonator arrangements reveals novel insights into the impact of disorder and irregularity on bandgap characteristics, enriching our understanding of metastructure dynamics. The robust validation of our findings through a combined numerical and experimental approach strengthens the credibility of our proposed metastructure design. The dual functionality of continuous beam resonators, discovered through our analysis, holds promising implications for the broader applicability of metabeam systems. Beyond merely suppressing vibrations, these systems exhibit the ability to harvest energy simultaneously, enhancing their practicality across diverse engineering applications. The significance of this work lies not only in its fundamental contributions to the field but also in the practical guidance it offers. Key contributions of the paper include:

- *Practical Metastructure Design:* The proposed continuous metabeams redefine the landscape of locally resonant metamaterials, offering a tangible and practical architecture that transcends traditional discrete resonator models.
- *Insights into Disorder Effects:* Our comprehensive investigation into uniform and non-uniform resonator arrangements, incorporating the concept of tacticity, unveils novel insights into the influence of disorder and irregularity on the bandgap characteristics of metastructures.
- *Tacticity-Inspired Design:* Leveraging inspiration from polymer science, our metabeam designs introduce innovative mass distribution concepts, providing fabrication-friendly methods to effectively modulate bandgap characteristics in metastructures. It is the first experimental study to understand the bandgap formation mechanism in tacticity-inspired metastructures design.
- *Validation through Numerical and Experimental Approaches:* The combined numerical and experimental methodology ensures the robust validation of bandgap phenomena in beam-based metastructures. Experimental results strongly corroborate the predictions from our simulations.
- *Dual Functionality Exploration:* Our analysis of continuous beam resonator responses reveals the promising dual functionality of metabeam systems - simultaneous vibration suppression and energy harvesting. This dual capability significantly broadens the applicability and potential impact of these systems.

Therefore, the inclusion of tacticity-based design has remarkable implications on the dispersive behavior of fabricated metabeams. The distribution of end masses on secondary beams and arrangements as isotactic or syndiotactic metabeams concedes to perceive interesting dynamic properties. To implement tacticity inspired design, we can think of a mechanical metastructure in which, on the cylindrical surface of a primary beam, we can use secondary beams as resonators with a capacity to slide circumferentially on the primary beam, thus practically realizing controllable tacticity. Equal end mass distribution for the isotactic uniform metabeam designs can be used for elastic wave attenuation in two different frequency bands without changing the stiffness and mass density of the metastructure. For unequal end mass distribution, isotactic and syndiotactic uniform metabeam designs can also facilitate elastic wave attenuation in two frequency bands. The equal and unequal combination of end masses gives a hybrid uniform metabeam design having a mixture of isotactic and syndiotactic configurations, extending elastic wave attenuation in multiple frequency bands. The width of these frequency bands can be increased by designing isotactic, syndiotactic, or hybrid non-uniform metabeams. Unlike tuned mass damper, which operates for a single resonance frequency, the tacticity-based metastructures can be used to target elastic wave attenuation in multiple frequency ranges. These locally resonant bandgaps can be tuned easily with the physical properties of resonators (mass and stiffness) and with the novel tacticity-based designs of mechanical metastructure. Hence, a tacticity-based design strategy for mechanical metastructures can be influential in all the research fields of vibrations, such as mechanical engineering, aerospace, and civil, for manipulating elastic waves due to consequential dynamic properties. The new findings and methodologies pave the way for advancements in locally resonant metamaterials, creating opportunities for further research, collaboration with material scientists, and optimization for varied engineering applications. In conclusion, our study not only bridges the gap between theory and application but also sets the stage for transformative impacts in the realm of engineered materials.

## Methods

## Materials

The parts of the metabeam (see supplementary Fig. S1) and fixtures are manufactured with aluminum. The material properties are characterized by Young’s Modulus $$E =68$$ GPa, Poisson’s ratio $$\nu =0.33$$, and density $$\rho = 2.7$$$$gm/cm^3$$. The end mass on secondary beams is fabricated with mild steel with material properties Young’s Modulus $$E =210$$ GPa, Poisson’s ratio $$\nu =0.30$$ and density $$\rho = 7.85$$$$gm/cm^3$$.

## Fabrication

The dimensions of metabeam parts and fixtures are delineated in the table (see supplementary table S1) and designed using SOLIDWORKS. The ASTM standard (E756-98) is followed in designing the metabeam, which suggests that including the root section in the metabeam is essential to get suitable and meaningful experimental measurements. It propounds that the thickness of the root section should be at least twice the thickness of the main beam. All parts of the metabeam are fabricated using conventional manufacturing methods. The profile of the primary and secondary beams is cut using the conventional water jet machining (WJM) process, and rectangular slots in the primary beam are cut by a non-traditional wire-cut electrical discharge machine (EDM). The end masses on the secondary beams are fabricated using the wire-cut EDM. The detailed information about each method with dimensions of each parts are given in the supplementary section 1.1.

### Experimental displacement transmittance

The schematic block diagram of the complete experimental set-up to perform contact-less dynamic vibration testing with the scanning Laser Doppler Vibrometer (LDV) and the actual experimental arrangement of all equipment used with detailed information is shown (see supplementary section 1.3) in Figs. S4 and S5, respectively. All the designed metabeams are tested using this technique for measuring displacement transmissibility with the help of the electrodynamic shaker (LDS V780) and 3D Laser Doppler Vibrometer (LDV PSV-400). Data acquisition and signal processing are done through the NI DAQ system. The endpoint and base of the metabeam are scanned with Polytec LDV to measure the displacements at these points. Hence, these locations are shielded with retro-reflective tape to enhance the reflection of the incident laser beam, which will help in more accurate measurements. All the displacement transmissibility measurements of the fabricated metabeams are done with the pseudo-random base excitation signal with 1600 FFT lines with the help of an electrodynamic shaker and function generator. The velocity decoder in the controller maps the voltage proportional to the velocity of the scanned point, which is measured by scanning Polytec LDV. While calculating the frequency response, the measured velocity is converted to displacement to obtain the displacement transmissibility ratio on a logarithmic scale.

### Numerical displacement transmittance

The finite element analysis simulations are carried out for all the fabricated metabeams in COMSOL Multiphysics. The frequency domain analysis is done with tetrahedral extremely fine physics-controlled mesh having 85616 domain elements, 38978 boundary elements, and 7914 edge elements for all the metabeams. The root section of the metabeam is given base excitation with an amplitude of 0.001 m in *z*-direction. A domain point probe is set to measure displacement at the end of the metabeam in *z*-direction. The displacement ratio at the endpoint to the root section gives displacement transmittance on a logarithmic scale, which is compared with experimentally obtained displacement transmittance. With linear elastic material assumption, the Rayleigh damping of 1–2 $\%$ about the first two eigen frequencies is applied to match the bandgaps and amplitudes closely.

## Supplementary Information

Supplementary Information.

## Data Availability

All relevant data are available from the corresponding author upon reasonable request, and/or are included within the main part and Supplementary Information.
