# Supplementary material for: Bandgap formation mechanism in tacticity inspired elastic mechanical metastructures
Source: Sci Rep. 2024 Oct 19;14:24611. doi: 10.1038/s41598-024-75462-6 (PMC11490600; doi:10.1038/s41598-024-75462-6)
Supplement: Supplementary file 1 — Supplementary Information. [file 41598_2024_75462_MOESM1_ESM.pdf]

Supplementary material: Bandgap formation mechanism in tacticity inspired elastic mechanical metastructures

Ankur Dwivedi[1,*], Rajendra Kumar Munian[2], Bishakh Bhattacharya[1], and Sondipon Adhikari[3]

[1]Department of Mechanical Engineering, Indian Institute of Technology Kanpur, India
[2]Department of Mechanical Engineering, Indian Institute of Technology Ropar, India
[3]James Watt School of Engineering, University of Glasgow, United Kingdom
[1]ankurdwi@iitk.ac.in

Contents

1 Supplementary Methods **1**
1.1 Design and manufacturing of metabeams . . . 1
1.2 Analytical analysis of the metabeam . . . 3
1.3 Experimental set-up for measuring the displacement transmittance . . . 6
1.4 Comparison of experimental and numerical displacement transmittance for the metabeams . . . 8
2 Supplementary Discussion **11**
References **12**

1 Supplementary Methods

1.1 Design and manufacturing of metabeams

This section explains the detailed design and manufacturing process used to fabricate the metabeams. Discrete structures are suitable for understanding the physical phenomenon but are challenging to implement practically. Based on the propagation characteristics exhibited by discrete locally resonant lattice structure described in[1,2] and later by implementing strategies to continuous systems like a beam attached with 3-dof double negative resonator[3], we endeavor to design such a continuous system and investigate the bandgap formation experimentally. The components of the metabeam, as shown in Figure S1, are designed using the SolidWorks by following the dimensions and material stated in Table S1. As portrayed in Figure S1 (a), the primary beam has two parts: the main beam and the root section. The primary beam is designed by following the ASTM standard (E756-98), which suggests that including the root section is essential in the design for suitable and meaningful experimental

Table S1. Dimensions and materials for the designed metabeams

Component	Material	Dimensions (mm)	
Primary Beam	Aluminium	***Main Beam***	***Root Section***
		Length=430	Length=80
		Width=20	Width=58
		Thickness=10	Thickness=20
Secondary Beam	Aluminium	Length=80<br>Width=5.0<br>Thickness=2.0	
End mass	Mild Steel	Length=Width=Thickness=5.0	
Circular Fixture	Aluminium	Diameter=100<br>Thickness=5.0	
Rectangular Fixture	Aluminium	Length=Width=80<br>Thickness=5.0	

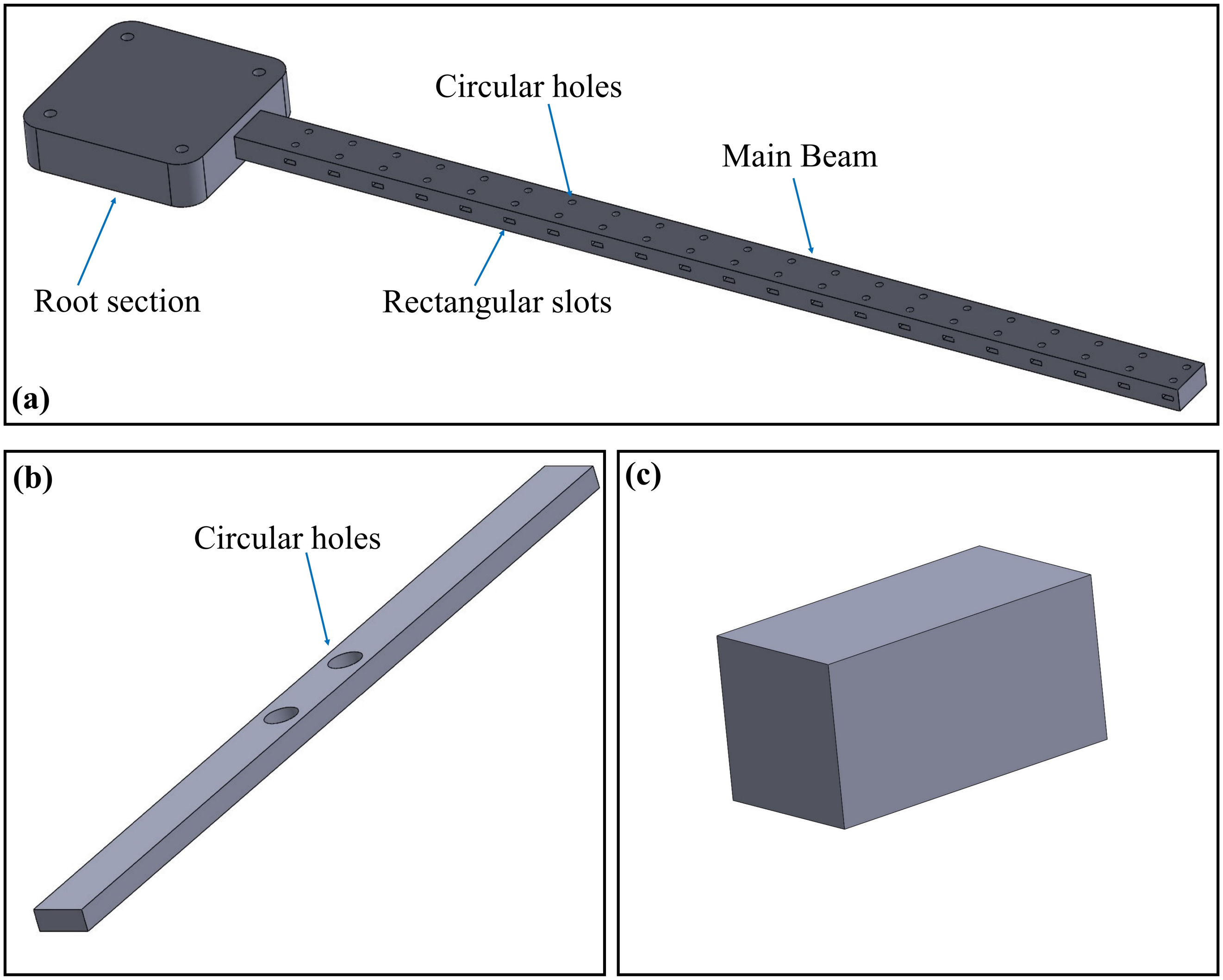

Figure S1. Part designs of the metabeam. (a) The primary beam has rectangular slots and circular holes for attaching the secondary beam as a local resonator. An extended root section is shown to attach the primary beam to the fixture. (b) The secondary beam has circular holes for attaching it to the primary beam. (c) The end mass has to be placed at both tips of the secondary beam.

measurements. The thickness of the root section should be at least twice the thickness of the main beam. Following these standards, experimental results can be obtained by satisfying the boundary conditions at the root section, which is fastened using the fixtures. Apart from this, the main beam has 21 rectangular slots with dimensions (5 mm $\times$ 2 mm) uniformly spaced at 20 mm from their center point. Figure S1 (b) and (c) show the secondary beam and end mass to be mounted on both tips, respectively. The profile of the primary and secondary beams are cut using the conventional water jet machining (WJM) process, and rectangular slots in the primary beam are cut by a non-traditional wire-cut electrical discharge machine (EDM). To attach the secondary beams to these rectangular slots, the circular through-holes are projected cross-way through the rectangular slots on the top surface of the main beam. The circular through-holes having a diameter of 3 mm are done at a distance of 35 mm from the fixed end of the primary beam to the center of a hole on the top mid-surface of the beam. The distance between the center of both holes is 10 mm. As the secondary beam is to be inserted in the primary beam, its design is replicated as rectangular slots in the primary beam. Hence, the secondary beams have a cross-section of (5 mm $\times$ 2 mm) as resonators are also placed inside rectangular slots of the primary beam (the dimension of the rectangular slot is the same as the cross-section of the secondary beam). The end masses on the secondary beams are fabricated using the wire-cut EDM. These end masses have dimensions (5 mm $\times$ 5 mm $\times$ 5 mm), and a through-hole of 2 mm diameter is done to fix these at both ends of the secondary beam.

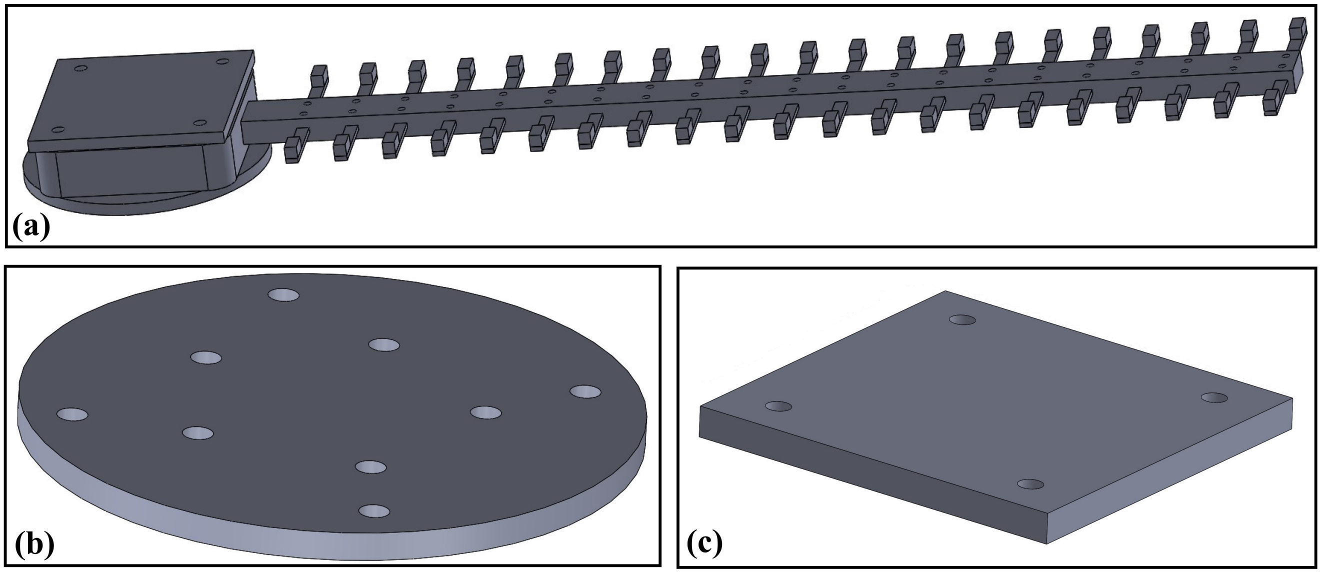

Figure S2. (a) Assembled metabeam having primary beam with attached secondary beams as a local resonator in rectangular slots. At both ends of secondary beams, end masses are attached. The extended root section can be mounted on the electro-dynamic shaker with the help of fixtures. (b) A lower circular fixture is attached to the electro-dynamic shaker. (c) The upper rectangular fixture sandwiches the primary beam with the lower circular fixture.

Figure S2 (a) shows assembled part designs as previously described in Figure S1. For mounting the designed metabeam on the electrodynamic shaker, fixtures are required, which are shown in Figure S2 (b) and (c). The circular fixture in Figure S2 (b) is attached to the electrodynamic shaker for transmitting the excitation to the vibrating metabeam. It has a standard design as per the shaker. It has five circular holes of 5 mm diameter at 50 pitch circle diameter (pcd), aligned at 72 degrees with each other. The outer four circular holes have a diameter of 6 mm and can be used to fasten the metabeam with the help of upper rectangular fixture shown in Figure S2 (c). The upper rectangular fixture is used to sandwich the metabeam with the lower circular fixture, as shown in Figure S2 (a).

1.2 Analytical analysis of the metabeam

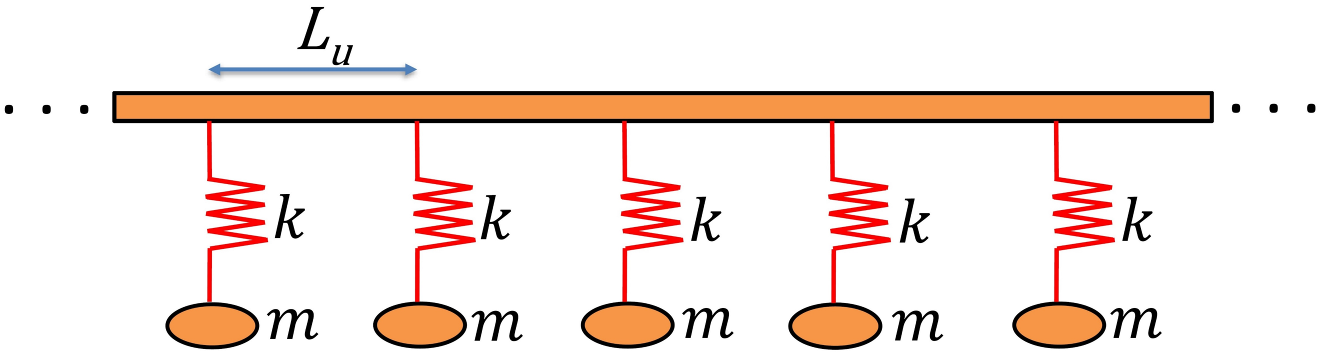

Figure S3. Equivalent design of the metabeam with attached discrete local resonators corresponding to continuous secondary beams as a resonator with end mass.

Figure S3 shows the equivalent design of the metabeam described in the main text (Figure 1 (a)). It has discrete local resonators corresponding to continuous resonators having mass (m) and stiffness (k). Based on the information given about the designed metabeam with the specified dimensions in Table S1, the unit cell of the metabeam will have slenderness ratio 2.0 ($L_u/t = 20/10 = 2.0$). It suggests formulating the equivalent metabeam with discrete resonators using Timoshenko beam theory.

Equation of motion of the Timoshenko beam for free vibration[4–6] can be written as

$$
\begin{aligned}
EI\frac{\partial^2\phi(z,t)}{\partial z^2}+GA\kappa\left(\frac{\partial w(z,t)}{\partial z}-\phi\right)-\rho I\frac{\partial^2\phi(z,t)}{\partial t^2}=0\\
GA\kappa\frac{\partial}{\partial z}\left(\frac{\partial w(z,t)}{\partial z}-\phi\right)-\rho A\frac{\partial^2 w(z,t)}{\partial t^2}=0
\end{aligned}
\tag{S1}
$$

where E, G, A, I, ρ and κ are the Young modulus, shear modulus, area, second moment of area, density of the material, and shear correction factor for the beam. w is the transverse deflection of the beam in the z-direction, ϕ is rotation due to pure bending, which are the function of the space (z) and time (t). The following expression can express the shear force and bending moment for the beam

$$
\begin{aligned}
V=GA\kappa\left(\frac{\partial w(z,t)}{\partial z}-\phi\right)\\
M=-EI\frac{\partial\phi(z,t)}{\partial z}
\end{aligned}
\tag{S2}
$$

After manupulations, Eq.(S1) can be condensed in a single partial differential equation[7] as

$$
EI\frac{\partial^4 w(z,t)}{\partial z^4}-\left(\frac{\rho EI}{G\kappa}+\rho I\right)\frac{\partial^4 w(z,t)}{\partial^2 z\partial^2 t}+\frac{\rho^2 I}{G\kappa}\frac{\partial^4 w(z,t)}{\partial t^4}+\rho A\frac{\partial^2 w(z,t)}{\partial t^2}=0 \tag{S3}
$$

Equation (S3) can be non-dimensionlized by assuming $z=L_u\tilde{z}$, $t=\omega_b\tau$, $w(z,t)=W\tilde{w}(\tilde{z},\tau)$, $\phi(z,t)=\frac{W}{L_u}\tilde{\phi}(\tilde{z},\tau)$. Here L_u is length of the unit cell of the metabeam, ω_b is the natural frequency of the beam. L_u, ω_b and W relate the respective dimensional terms to non-dimensional terms. Based on this non-dimensional form of Timoshenko beam equation as follows

$$
\frac{\partial^4\tilde{w}(\tilde{z},\tau)}{\partial\tilde{z}^4}-\psi_1\frac{\partial^4\tilde{w}(\tilde{z},\tau)}{\partial^2 z\partial^2\tau}+\psi_2\frac{\partial^4\tilde{w}(\tilde{z},\tau)}{\partial\tau^4}+\frac{\partial^2\tilde{w}(\tilde{z},\tau)}{\partial\tau^2}=0 \tag{S4}
$$

where ${\omega_b}^2=\frac{EI}{\rho AL_u^4}$, $\psi_1=\left(\frac{\rho E}{G\kappa}+\rho\right)\frac{{\omega_b}^2L_u^2}{E}=(\delta_m+1)\,\delta_g$, $\psi_2=\frac{\rho^2L_u^4}{EG\kappa}{\omega_b}^4=\delta_m\delta_g^2$.

$\delta_m=\frac{E}{G\kappa}$ and $\delta_g=\frac{I}{AL_u^2}$ are the material and geometric parameters. For solving Eq. (S4), we assumed the displacement $\tilde{w}(\tilde{z},\tau)=X(\tilde{z})e^{i\overline{\Omega}\tau}$, where $\overline{\Omega}=\frac{\varpi}{\omega_b}$ is the ratio of the temporal frequency of the wave to the natural frequency of the beam. After substituting this form of the displacement in Eq.(S4), Timoshenko beam equation could be rewritten as

$$
X^{IV}(\tilde{z})+\psi_1\overline{\Omega}^2X^{II}(\tilde{z})+(\psi_2\overline{\Omega}^4-\overline{\Omega}^2)X(\tilde{z})=0 \tag{S5}
$$

Further, the dimensionless slope, bending moment and shear force[4] can be written as

$$
\begin{aligned}
\phi(z)=\frac{1}{a_2}X^{III}-\frac{a_1}{a_2}X^{I}\\
M(z)=-\phi(z)^{I}=-X^{II}(z)-\alpha X(z)\\
V(z)=\phi(z)^{II}=\beta_1X^{I}(z)-\beta_2X^{III}(z)
\end{aligned}
\tag{S6}
$$

where $a_1=\frac{1}{\delta_m\delta_g}-\delta_m\delta_g\overline{\Omega}^2$, $a_2=\delta_g\overline{\Omega}^2-\frac{1}{\delta_m\delta_g}$, $\alpha=\delta_m\delta_g\overline{\Omega}^2$, $\beta_1=(1+\frac{a_1}{a_2})$, $\beta_2=\frac{1}{a_2}$.

The mode shape function $X(\tilde{z}$ for the Timoshenko beam to obtain the general solution can be assumed as

$$
X(\tilde{z})=\begin{bmatrix}\cos(\lambda\tilde{z}) & \sin(\lambda\tilde{z}) & \cosh(\lambda\tilde{z}) & \sinh(\lambda\tilde{z})\end{bmatrix}\times\underbrace{\begin{bmatrix}A & B & C & D\end{bmatrix}^T}_{Constants} \tag{S7}
$$

where A,B,C, and D are unknown constants. The defined set of boundary conditions can find these constants. Substitution of the assumed solution from Eq.(S7) in Eq.(S5) yields the value of eigenfrequency λ for Timoshenko beam as $\lambda^2=\mp\frac{\psi_1\overline{\Omega}^2}{2}\pm\sqrt{\frac{\psi_1^2\overline{\Omega}^4-4(\psi_2\overline{\Omega}^4-\overline{\Omega}^2)}{2}}$.

The continuity equations for the nth unit in the metabeam can be written for displacement, slope, moment and shear force, respectively as

$$\begin{aligned}
& w_n(0,t) = w_{n-1}(L_b,t) \rightarrow \tilde{w}_n(0,\tau) = \tilde{w}_{n-1}(1,\tau) \rightarrow X_n(0) = X_{n-1}(1) \\
& \frac{\partial w_n}{\partial z}\Big|_0 = \frac{\partial w_{n-1}}{\partial z}\Big|_1 \rightarrow \tilde{w}_n^I(0,\tau) = \tilde{w}_{n-1}^I(1,\tau) \rightarrow X_n^{II}(0) = X_{n-1}^{II}(1) \\
& \tilde{\phi}_n^I(0,\tau) = \tilde{\phi}_{n-1}^I(1,\tau) \rightarrow X_n^{III}(0) - a_1 X_n^I(0) = X_{n-1}^{III}(1) - a_1 X_{n-1}^I(1) \\
& \frac{\partial^2 w_n}{\partial z^2}\Big|_0 = \frac{\partial^2 w_{n-1}}{\partial z^2}\Big|_1 \rightarrow \tilde{w}_n^{II}(0,\tau) = \tilde{w}_{n-1}^{II}(1,\tau) \rightarrow M_n(0) = M_{n-1}(1) \\
& \rightarrow -X_n^{II}(0) - \alpha X_n(0) = -X_{n-1}^{II}(1) - \alpha X_{n-1}(1) \\
& \frac{\partial^3 w_n}{\partial z^3}\Big|_0 - \frac{f_n e^{i\overline{\omega}t}}{EI}\Big|_0 = \frac{\partial^3 w_{n-1}}{\partial z^3}\Big|_1 \rightarrow \tilde{w}_n^{III}(0,\tau) - \frac{\tilde{f}_n {L_b}^3 e^{i\overline{\Omega}\tau}}{WEI}\Big|_0 = \\
& \tilde{w}_{n-1}^{III}(1,\tau) \rightarrow \beta_1 X_n^I(0) + \beta_2 X_n^{III}(0) - \mu_f = \beta_1 X_{n-1}^I(1) + \beta_2 X_{n-1}^{III}(1)
\end{aligned} \tag{S8}$$

where f_n is the amplitude of the shear force induced to the system due to the discrete oscillator attached to the beam.

The shear force due to the oscillator on the beam can be found by the free body analysis of the system for n^{th} oscillator along z direction. It gives the shear force as

$$f_n = wk\left[\frac{\overline{\Omega}^2}{\Omega^2 - \overline{\Omega}^2}\right] \tag{S9}$$

where $\Omega = \frac{\omega}{\omega_b}$ is the ratio of the natural frequency of the resonators (m,k) to the natural frequency of the beam. Now, all the continuity conditions can be non-dimensionalized as done using the displacement in Eq.(S4). The non-dimensionalized term in the shear force continuity equation can be further simplified as

$$\mu_f = \frac{\tilde{f}_n {L_b}^3}{WEI} = \underbrace{\frac{k{L_b}^3}{EI}}_{\mu_s}\underbrace{\left[\frac{\overline{\Omega}^2}{\Omega^2 - \overline{\Omega}^2}\right]}_{\chi} X_n(0) = \mu_s \chi X_n(0) = \vartheta X_n(0) \tag{S10}$$

where $\vartheta = \mu_s \chi$.

Therefore, all the continuity conditions in Eq.(S8) can be condensed in the form of a matrix as:

$$\begin{aligned}
& \begin{bmatrix} 1 & 0 & 0 & 0 \\ 0 & -a_1 & 0 & 1 \\ \alpha & 0 & 1 & 0 \\ -\vartheta & \beta_1 & 0 & \beta_2 \end{bmatrix} \begin{Bmatrix} X_n(0) \\ X_n^I(0) \\ X_n^{II}(0) \\ X_n^{III}(0) \end{Bmatrix} = \begin{bmatrix} 1 & 0 & 0 & 0 \\ 0 & -a_1 & 0 & 1 \\ \alpha & 0 & 1 & 0 \\ 0 & \beta_1 & 0 & \beta_2 \end{bmatrix} \begin{Bmatrix} X_{n-1}(1) \\ X_{n-1}^I(1) \\ X_{n-1}^{II}(1) \\ X_{n-1}^{III}(1) \end{Bmatrix} \\
& \rightarrow \underbrace{\begin{bmatrix} 1 & 0 & 1 & 0 \\ 0 & -a_1\lambda - \lambda^3 & 0 & -a_1\lambda + \lambda^3 \\ \alpha - \lambda^2 & 0 & \alpha + \lambda^2 & 0 \\ -\vartheta & \beta_1\lambda - \beta_2\lambda^3 & -\vartheta & \beta_1\lambda + \beta_2\lambda^3 \end{bmatrix}}_{K} \underbrace{\begin{Bmatrix} A_n \\ B_n \\ C_n \\ D_n \end{Bmatrix}}_{\Lambda_n} = \\
& \underbrace{\begin{Bmatrix} \cos\lambda & \sin\lambda & \cosh\lambda & \sinh\lambda \\ -\lambda\sin\lambda & \lambda\cos\lambda & \lambda\sinh\lambda & \lambda\cosh\lambda \\ -\lambda^2\cos\lambda & -\lambda^2\sin\lambda & \lambda^2\cosh\lambda & \lambda^2\sinh\lambda \\ \lambda^3\sin\lambda & \lambda^3\cos\lambda & \lambda^3\sinh\lambda & \lambda^3\cosh\lambda \end{Bmatrix}}_{H} \underbrace{\begin{Bmatrix} A_{n-1} \\ B_{n-1} \\ C_{n-1} \\ D_{n-1} \end{Bmatrix}}_{\Lambda_{n-1}}
\end{aligned} \tag{S11}$$

This gives

$$\Lambda_n = \mathrm{K}^{-1}\mathrm{H}\Lambda_{n-1} \tag{S12}$$

From Bloch-Floquet's theorem, we have the relationship between the state vectors of the successive unit cell as:

$$\phi_n(1) = e^{-\mu} \cdot \mathbf{I} \cdot \phi_{n-1}(1) \tag{S13}$$

where I is an identity matrix of size 4×4, ϕ is the state vector and μ is non-dimensional wave number.

Using Eqns.(S12) and (S13) we can deduce following expression

$$\begin{aligned} &\phi_n(1) = \mathrm{H}\Lambda_n = \mathrm{HK}^{-1}\mathrm{H}\Lambda_{n-1} = \underbrace{\mathrm{HK}^{-1}}_{T}\phi_{n-1}(1) \\ &\rightarrow |\mathrm{T} - e^{-\mu}\cdot \mathrm{I}|\phi_{n-1}(1) = 0 \rightarrow e^{-\mu} = \underbrace{\mathrm{eig(T)}}_{u} \rightarrow \mu = ln(u) \end{aligned} \tag{S14}$$

From the mathematical expression derived in Eqn.(S14), it can be concluded that the wave number is a function of the logarithmic of the eigenvalues of the transfer matrix. Therefore, the band structure of the metabeam can be plotted using this relation for a periodic unit-cell metastructure. Moreover, while performing the experimental investigation of bandgap, due to the effects of non-periodic constructions of the metabeam and boundary conditions of the finite structures, the experimentally demonstrated displacement transmissibility can not be well validated with conventional unit cell based dispersion theory, which is developed for infinite periodic structures. Hence, a comprehensive numerical analysis of the vibration characteristics of the metabeams can be done using a well-established finite element software package.

1.3 Experimental set-up for measuring the displacement transmittance

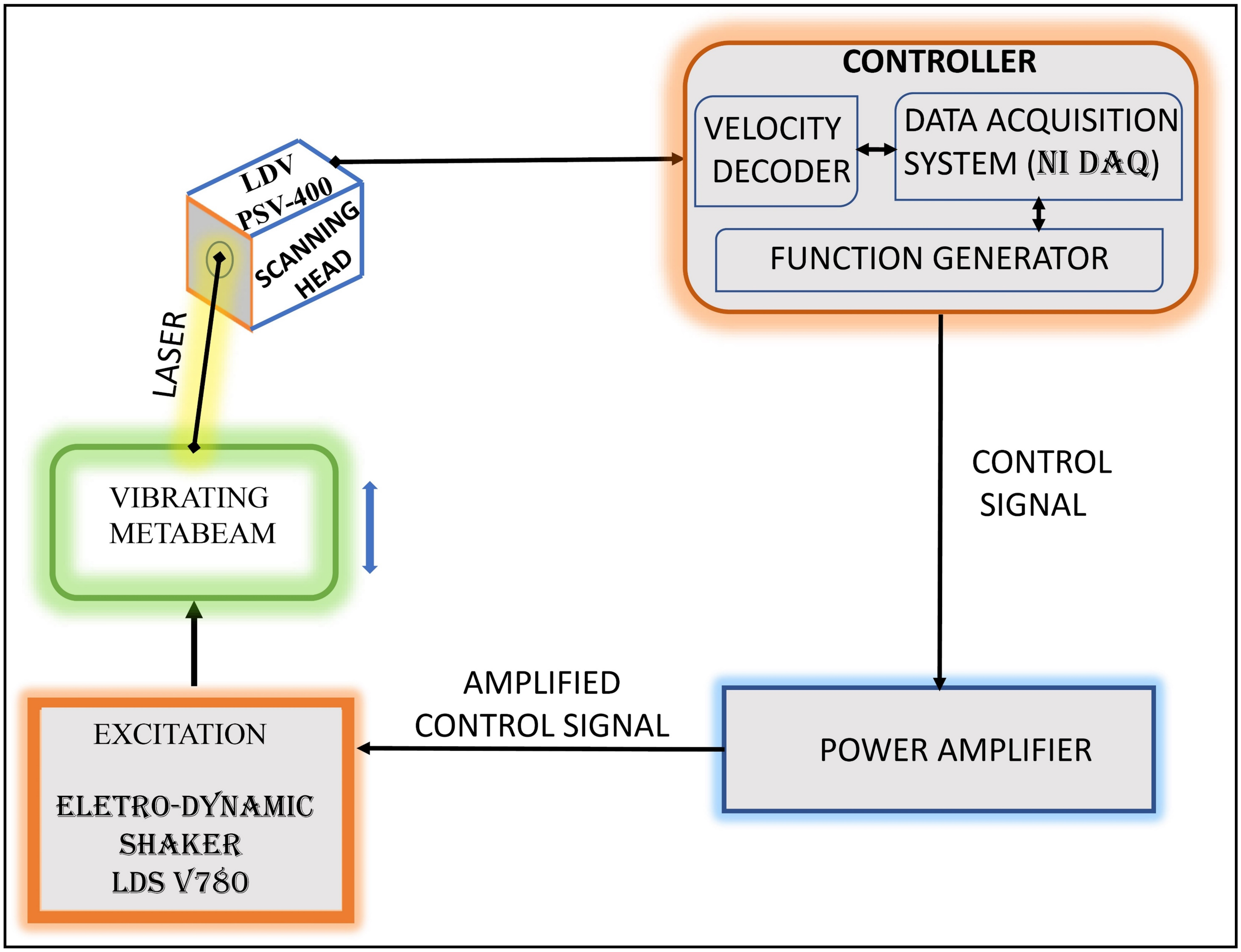

Figure S4. Schematic block diagram of the experimental set-up describing the different equipment involved in measuring displacement transmissibility of the metabeams for contact-less dynamic vibration testing.

In this section the detailed experimental procedure to obtain displacement transmittance is described. Figure S4 shows the schematic block diagram of the experimental set-up to perform contact-less dynamic vibration testing on the designed

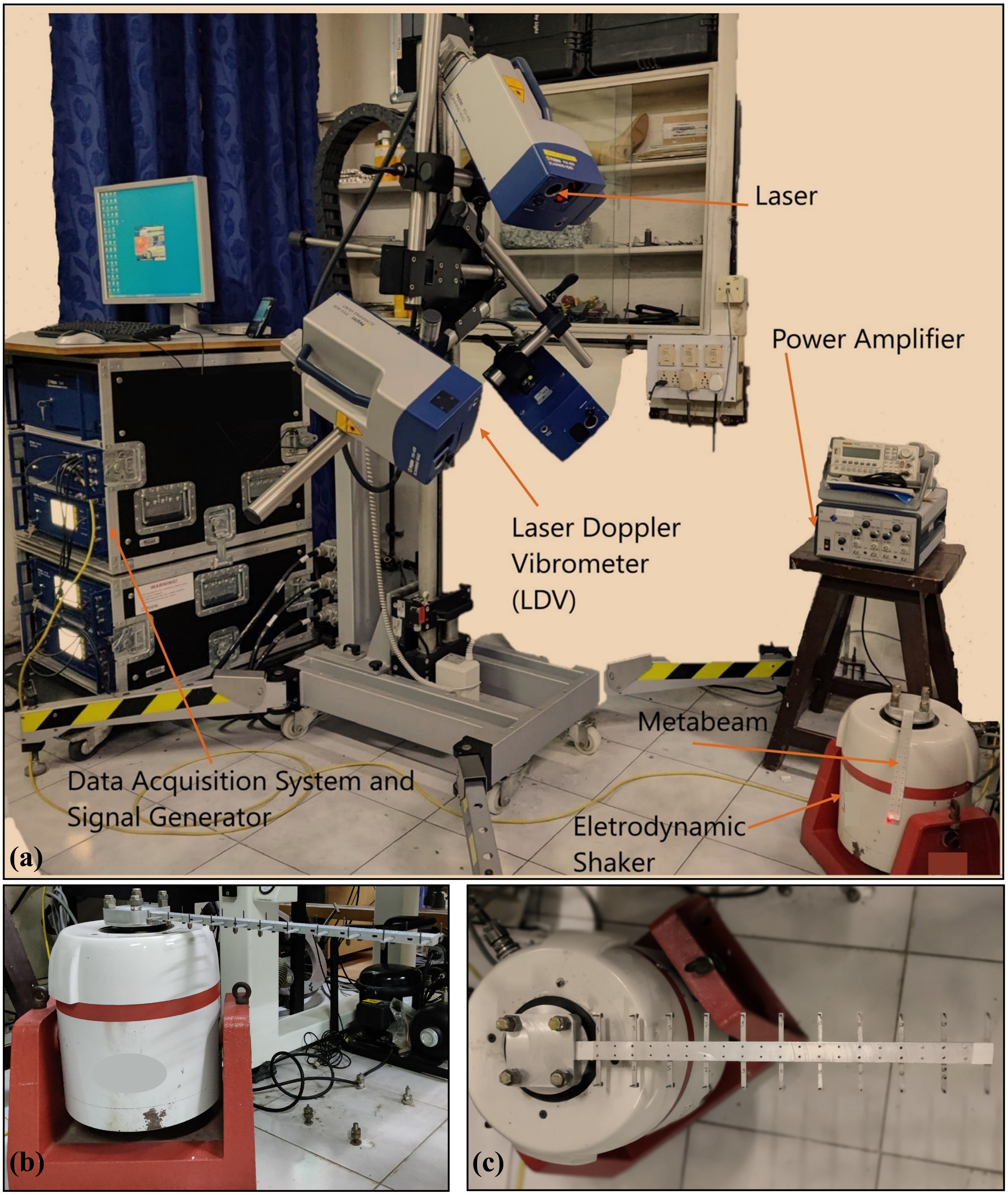

Figure S5. (a) Experimental arrangement describes the measurement of displacement transmissibility for the designed metabeam mounted on the electrodynamic shaker. The endpoint and base of metabeam is scanned with the help of a 3D scanning Laser Doppler Vibrometer (LDV). The metabeam is given base excitation with a pseudo-random signal. (b) Closer view of metabeam having primary beam with attached end mass loaded secondary beams, mounted on the electrodynamic shaker with fixture attachments. (c) The top view of the metabeam shows secondary beam attachments to the primary beam with the end masses on the secondary beams more clearly.

metabeams for measuring displacement transmissibility. It provides information about equipment used to experimentally obtain the displacement transmittance for the metabeams. Figure S5 (a) portrays the actual experimental arrangement of all equipment used in the laboratory for measuring the displacement transmissibility of the designed metabeams. Figure S5 (b) shows the metabeam mounted on the electrodynamic shaker (LDS V780). From the top view, as shown in Figure S5 (c), the metabeam having secondary beam attachments with tip masses at both ends can be visualized more clearly. The selection of the appropriate excitation signal is vital for quality vibration testing. The linearity of the testing sample should also be considered while choosing the excitation signal. The random signal should be preferred for the linear approximation of a non-linear testing sample. Therefore, to measure the displacement transmissibility of the metabeam, it is given a pseudo-random base excitation signal with 1600 FFT lines with the help of an electrodynamic shaker and function generator. All FFT lines of the measured frequency spectrum are excited simultaneously with phase differences, generated using a uniformly distributed random number. Data acquisition and signal processing are done through the NI DAQ system. The control signals can be amplified by setting different gains in the power amplifier. The amplified control signal can be fed to the electrodynamic shaker for vibrating the mounted metabeams as shown in Figure S4. The endpoint and base of the metabeam are scanned with a 3D Laser Doppler Vibrometer (LDV PSV-400). Hence, these locations are shielded with retro-reflective tape to enhance the reflection of the incident laser beam, which will help in more accurate measurements. The velocity of the marked scanned points is measured with scanning Polytec LDV. The velocity decoder in the controller maps the voltage proportional to the velocity of the scanned point. While calculating the frequency response, the measured velocity is converted to displacement for obtaining the displacement transmissibility ratio.

1.4 Comparison of experimental and numerical displacement transmittance for the metabeams

In this section, bandgap formation in a vibrating metabeam is investigated numerically and experimentally with the help of the displacement transmittance response of the system for the uniform and non-uniform metabeam configurations. The uniform metabeam has 11 secondary beams (acting as local resonators) attached equidistant from each other. In contrast, the non-uniform metabeam has a random attachment of 11 secondary beams in the primary beam. The main text describes exact configuration of the uniform and non-uniform metabeams having details of secondary beams attachments to the primary beam in figures 1c and 1d, respectively. The schematic for each uniform metabeam with details of end mass distribution is shown in the respective panels. We first see the effect of secondary beam attachments embedded in a simple beam to configure uniform and non-uniform configurations. Later, the effect of increasing end mass and the number of resonators on the bandgap formation is also explored for the uniform and non-uniform metabeam configurations. Further, as described already in the main text one more hybrid syndiotactic metabeam configuration based on tacticity is investigated for the uniform and non-uniform metabeam configurations. The following subsections explain the vibration characteristics of above mentioned uniform and non-uniform metabeam configurations in detail.

Uniform Metabeam

Figure S6 describes the displacement transmissibility responses for the uniform metabeam, which has 11 secondary beam attachments at constant distances from each other in the primary beam. Due to the attached secondary beams in the primary beam, the degree of freedom of the metabeam increases. It introduces additional eigen frequencies in a bandwidth. These secondary beams acting as local resonators help in attenuating the elastic waves through the metabeam by forming locally resonant bandgaps. The elastic waves having a frequency close to eigen frequencies of the secondary beams are absorbed in these bandgaps. Here, in Figure S6 (a), we demonstrate it by comparing the experimental displacement transmittance of the a simple beam with uniform metabeam. On the x-axis, the frequency in Hz is represented, and on the y-axis, the displacement ratio of the endpoint to the base of the beam is defined on a logarithmic scale. The uniform metabeam has secondary beams acting as resonators, whereas the simple beam has no resonators. The black curve shows the experimental displacement transmittance of the simple beam without any resonator. The natural frequency for the first mode is 296 Hz. On the other hand, the blue curve shows experimental displacement transmittance of uniform metabeam having 11 secondary beams acting as local resonators embedded at equal distances from each other in the primary beam. All the secondary beams have end masses of magnitude **m**, placed at both tips. The wide frequency regions in which the displacement transmittance response is negative exhibit the existence of attenuation bands. In the frequency spectrum of 544-572 Hz, 616-640 Hz, and 646-712 Hz, the displacement transmittance of the metabeam is negative in a wide frequency range. It indicates the formation of attenuation bands due to the suppressed propagation of elastic waves in these frequency regions. Hence, from the displacement transmittance response of the metabeam, it is evident that the secondary beam attachments acting as a local resonator help in forming locally resonant bandgaps, which are not present in the displacement transmittance response of a simple beam. Now, after observing bandgap formation with integration of secondary beams in primary beam to design a metabeam, we compare the numerical and experimental transmittance response for the fabricated uniform metabeam. Here, the black and blue curves show the numerical and experimental displacement transmissibility response of the metabeams, respectively. In Figure S6 (b) for the uniform metabeam, all the secondary beams have end masses of magnitude **m**, placed at both tips. The experimental

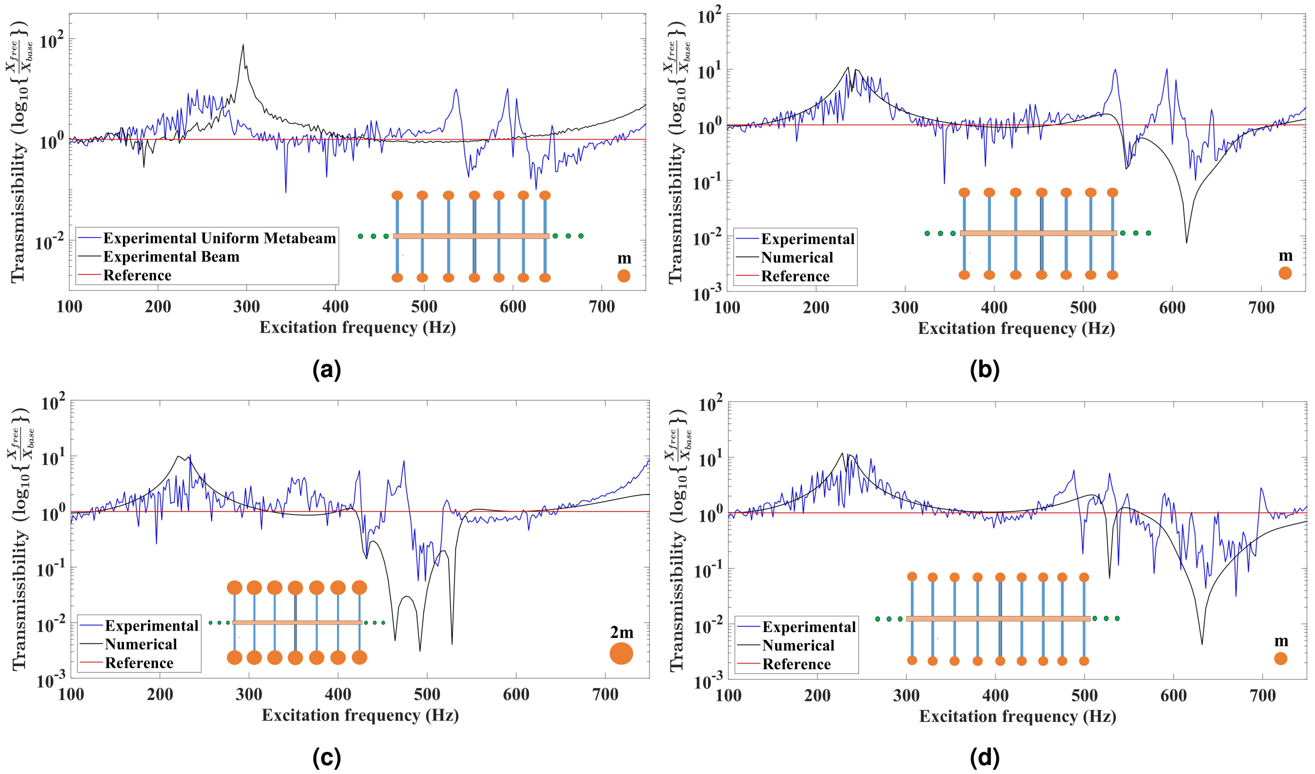

Figure S6. Comparison of the experimental displacement transmittance response for a beam with uniform metabeam. (a) The simple beam has no secondary beam attachments, whereas the uniform metabeam has **11** secondary beam attachments uniformly in the primary beam at a fixed distance from each other. The secondary beams at both ends have a tip mass **m**. Comparison of the experimental and numerical displacement transmittance response for the uniform metabeam. The primary beam has **11** secondary beams embedded uniformly at a fixed distance from each other to form the uniform metabeam configuration. The secondary beams at both tips have end mass (b) **m**. (c) **2m**. (d) The uniform metabeam with **20** secondary beams acting as resonators having end mass **m** at tips.

displacement transmittance response shows the attenuation bandgaps in the frequency spectrum of 544-572 Hz, 616-642 Hz, and 646-712 Hz, whereas the numerical displacement transmittance response shows bandgaps in frequency regions 542-712 Hz. Now, we see the effect of increased end mass in the resonators by keeping the number of secondary beams unchanged. As expected, in Figure S6 (c), the attenuation band shifts to the left on the frequency spectrum when the end masses at the secondary beams are increased to **2m**. It happens due to decreased natural frequencies of attached secondary beams as the end mass increases. The experimental displacement transmittance response shows the attenuation bandgaps in the frequency spectrum of 426-452 Hz, 480-518 Hz, and 528-544 Hz, whereas the numerical displacement transmittance response shows bandgaps in frequency regions 420-548 Hz. In contrast, an increase in the number of attached secondary beams in the primary beam to 20 shows a shifting of the attenuation band towards the right on the frequency spectrum as shown in Figure S6 (d). The experimental displacement transmittance response shows the attenuation bandgaps in the frequency spectrum of 556-588 Hz and 652-696 Hz, whereas the numerical displacement transmittance response shows bandgaps in frequency regions 520-548 Hz and 564-692 Hz. Some additional modes are excited at the frequencies 600 Hz, 620 Hz, and 644 Hz due to more added mass in the system, as the number of resonators is nearly double now. It implies that more resonators are required to attain attenuation in higher frequency regions to target higher modes. Hence, the optimal number of resonators increases with a targeted modal neighborhood for a fixed mass ratio.

Non-uniform Metabeam

As delineated through displacement transmissibility response in Figure S7, we now investigate the above-considered cases for the non-uniform metabeam, which has 11 secondary beams embedded in the primary beam at unequal distances from each other, acting as resonators. In Figure S7 (a), we are comparing the experimental displacement transmittance of a simple beam with the non-uniform metabeam. The simple beam has no resonators, whereas the non-uniform metabeam has secondary beam attachments acting as resonators. All the secondary beams have end masses of magnitude **m**, placed at both tips. The black

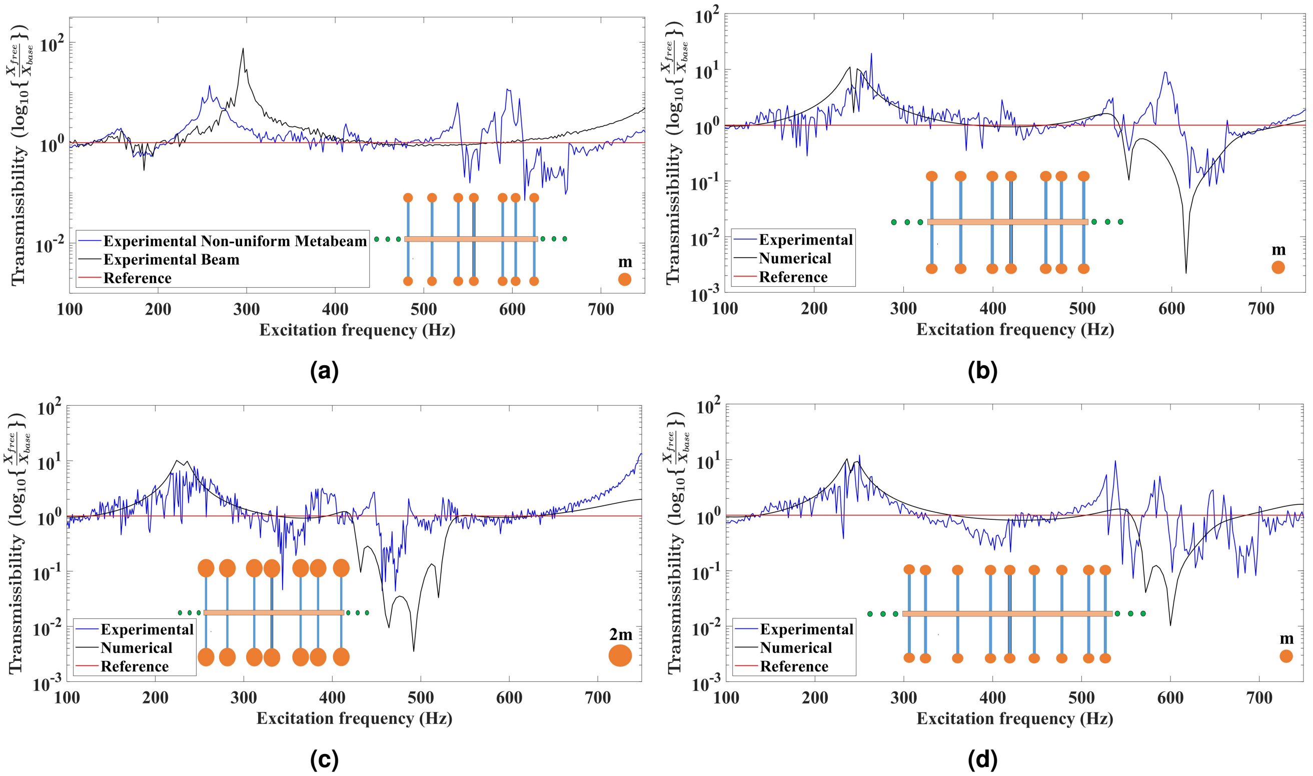

Figure S7. Comparison of the experimental displacement transmittance response for a beam with non-uniform metabeam. (a) The simple beam has no secondary beam attachments, whereas the non-uniform metabeam has random attachments of **11** secondary beam in the primary beam at a varying distance from each other. The secondary beams at both ends have a tip mass **m**. Comparison of the experimental and numerical displacement transmittance response for the non-uniform metabeam. The primary beam has **11** secondary beams embedded non-uniformly at varying distances from each other to form the non-uniform metabeam configuration. The secondary beams at both tips have end mass (b) **m**. (c) **2m**. (d) The non-uniform metabeam with **14** secondary beams acting as resonators having end mass **m** at tips.

and blue curves show the experimental displacement transmittance of simple beam and non-uniform metabeam, respectively. The natural frequencies for the first mode are 296 Hz and 258 for the simple beam and non-uniform metabeam respectively. As discussed earlier, here again we observe attenuation in frequency regions 544-564 Hz and 612-664 Hz due to inclusion of secondary beams. Hence the secondary beam attachments acting as a local resonator facilitate formation of locally resonant bandgaps, which are not observed in the displacement transmittance response of a simple beam. Now we compare the numerical and experimental displacement transmittance response for the fabricated non-uniform metabeam. Here, the black and blue curves show the numerical and experimental displacement transmissibility response of the metabeams, respectively. Figure S7 (b) depicts the non-uniform metabeam in which all the secondary beams have end masses of magnitude **m**, placed at both tips. The experimental displacement transmittance response shows the attenuation bandgaps in the frequency spectrum of 536-556 Hz and 608-660 Hz, whereas the numerical displacement transmittance response shows bandgaps in frequency regions 540-668 Hz. Some additional modes are excited with peaks at 546 Hz and 594 Hz in the experimental displacement transmittance response. When we increase the end masses to **2m** at the tip of the secondary beam, the attenuation band shifts to the left on the frequency spectrum as in Figure S7 (c). The shift in the bandgap frequency region is due to a decrease in the natural frequencies of secondary beams by increasing end mass in the system. The experimental displacement transmittance response shows the attenuation bandgaps in the frequency spectrum of 452-484 Hz and 504-518 Hz, whereas the numerical displacement transmittance response shows bandgaps in frequency region 448-524 Hz. Here again, additional modes are excited with peaks at 447 Hz and 487 Hz in the experimental displacement transmittance response. Further, in Figure S7 (d) the attenuation band shifts to the right on the frequency spectrum for the increased number of secondary beams to 14 in the primary beam. The experimental displacement transmittance response shows the attenuation bandgaps in the frequency spectrum of 552-568 Hz, 592-638 Hz, and 648-700 Hz, whereas the numerically simulated displacement transmittance response shows bandgaps in frequency region 556-692 Hz. The additional modes are again excited here in the experimental displacement transmittance response, having peaks at 586 Hz, 610 Hz, and 658 Hz. Hence, introducing more resonators shifts the frequency bandgap to the

right, which is beneficial if we want bandgap formation in a higher frequency region. Therefore, there is good agreement in experimental and numerically simulated displacement transmittance response for the non-uniform metabeams. However, we observe a slight decrease in the bandwidth compared to uniform metabeams due to changes in the eigen frequencies of the system. In contrast to uniform metabeams for an increased number of resonators, approximately the same frequency bandwidth can be obtained with fewer resonators for the non-uniform metabeam.

Hybrid metabeams

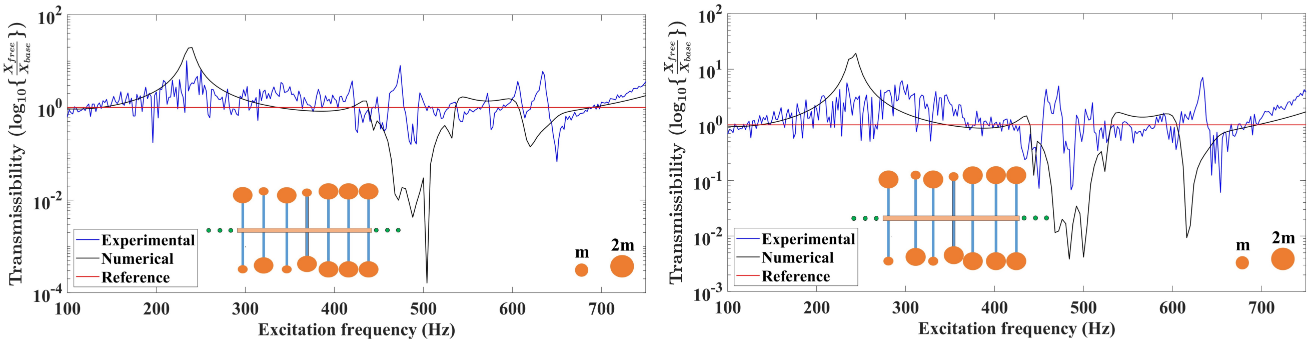

(a) (b)

Figure S8. Comparison of the experimental and numerical displacement transmittance response for the uniform and non-uniform hybrid metabeams based on tacticity. (a) For the uniform metabeam, the syndiotactic array in which end mass distribution of ***2m-m*** and ***m-2m*** is present on one side of the highlighted secondary beam and on another side ***2m-2m*** end mass distribution is present for isotacticity. (b) For the non-uniform metabeam, syndiotactic array in which end mass distribution of ***2m-m*** and ***m-2m*** is present on one side of the highlighted secondary beam and on another side ***2m-2m*** end mass distribution is present for isotacticity.

Figure S8 describes fabricated hybrid metabeam for the uniform and non-uniform configuration based on tacticity. For the uniform metabeam configuration, as shown in Figure S8 (a) on the left side of the highlighted secondary beam ***2m-m*** and ***m-2m*** end mass distribution is present to follow syndiotactic array and on right side ***2m-2m*** end mass distribution is present for isotacticity. The experimental displacement transmittance response shows the attenuation bandgaps in the frequency spectrum of 424-440 Hz, 450-460 Hz, 478-496 Hz, 504-532 Hz, 540-564 Hz, 576-588 Hz, 612-620 Hz, and 640-690 Hz. Due to additional excitation, there are peaks at frequencies of 474 Hz and 636 Hz in bandgaps. On the other hand, the numerical displacement transmittance response shows bandgaps in frequency regions 448-524 Hz and 620-684 Hz. The metabeam has only two types of secondary beams, so there are only two resonant bandgaps in numerical transmittance response. For the non-uniform metabeam configuration, in Figure S8 (b) the secondary beams have end mass distribution of ***2m-m*** and ***m-2m*** on left side of the highlighted mid secondary beam to follow syndiotactic array and ***2m-2m*** end mass distribution on right side for isotacticity. The experimental transmittance response shows the attenuation bandgaps in the frequency spectrum of 420-452 Hz, 476-492 Hz, 512-544 Hz, 556-580 Hz, 596-612 Hz and 640-684 Hz. There are peaks at frequencies 472 Hz, 510 Hz, and 636 Hz due to additional excited modes. The numerical transmittance response shows bandgaps in frequency regions 452-520 Hz and 606-690 Hz. We observe that inclusion tacticity results in a broader attenuation bandwidth than uniform metabeam in the non-uniform configurations.

2 Supplementary Discussion

This work not only addresses a pertinent problem in understanding bandgap formation but also provides a foundation for innovative designs with practical implications in various engineering applications. We proposed a beam-based metastructure design based on tacticity and investigated the bandgap formation mechanism through vibration characteristics to implement the findings of numerical studies and experimental studies in the actual field applications. These continuous secondary beams, acting as local resonators, extend beyond discrete resonators. The scanning Laser Doppler Vibrometer (LDV) is employed to experimentally capture the displacement transmissibility response within a frequency band, providing insights into the bandgap formation. The combined numerical and experimental methodology enables robust validation of bandgap phenomena in beam-based metastructures. The experiments strongly reinforce the simulation predictions. The design of continuous metabeams with embedded beam resonators provides a practical and realistic architecture for locally resonant metamaterials. It goes beyond idealized discrete resonator models[8–10]. We discussed the detailed design, manufacturing methods, and experimental setup to

obtain the displacement transmissibility response of metabeams. We analyzed metabeams having secondary beams attached to the primary beam acting as local resonators for the uniform and non-uniform configurations. The uniform metabeams, which have an arrangement of 11 secondary beams at a fixed distance from each other across the length of the primary beam, have been explored. Successively, the similar design of metabeams for the non-uniform configuration, having an arrangement of 11 secondary beams at varying distances across the length of the primary beam, has also been investigated. From the displacement transmissibility response, the bandgap formation can be seen due to the attachment of secondary beams acting as local resonators to the primary beam. We analyze the impact of adding end masses and increasing the number of secondary beams (resonators) on the formation of the attenuation bandgap for both configurations. In the uniform metabeam configuration, the bandgap shifts to the left by increasing the end mass on the secondary beam, which leads to an attenuation band in the lower frequency region. In contrast, an increased number of secondary beams in the uniform metabeam configuration shifts the bandgap to the right, forming the attenuation bands in the higher frequency region. We observed a similar bandgap shift by increasing end mass and the number of resonators for the non-uniform metabeam configuration. However, the attenuation bandwidth is smaller compared to uniform metabeams. To counter this, we further explored the metabeam designs based on tacticity for the uniform and non-uniform metabeam configurations as explained in the main text. The design of metabeams based on tacticity inspired from polymer science using innovative mass distribution concepts introduces new fabrication-friendly ways to modulate bandgap characteristics in metastructures. The distribution of end masses on secondary beams gives isotactic and syndiotactic metabeams designs. These metabeams designs modulate elastic wave propagation in the proposed metastructure in a feasible way to manifest interesting bandgap characteristics. While investigating uniform and non-uniform metabeam configurations by considering tacticity in the design, a wider attenuation band is reported in the case of non-uniform metabeams compared to uniform metabeams for equal or unequal end masses on either side of the secondary beam. Interestingly, isotactic and syndiotactic metabeam designs in uniform configuration can swap the locally resonant bandgaps without changing the natural frequencies of the secondary beams. The comprehensive investigation of uniform and non-uniform resonator arrangements with inclusion of tacticity reveals new insights into how disorder and irregularity can influence bandgap characteristics of metastructures. In non-uniform metabeam configurations, tacticity-based designs facilitate the formation of wider bandgaps, hence showcasing the potential for unconventional bandgap modulation. Lastly, we also explored the response of secondary beams to find the energy harvesting possibilities in the system. The continuous beam resonator response analysis elucidates the promising prospects of simultaneous vibration suppression and energy harvesting in metabeam systems. This dual functionality significantly enhances the applicability. We observed that the secondary beams vibrate with higher displacement amplitude in the bandgap frequency region, which could be feasible for harvesting vibrational energy with smart materials, hence enhancing the functionality of the system.

References

1. Dwivedi, A., Banerjee, A. & Bhattacharya, B. Simultaneous energy harvesting and vibration attenuation in piezo-embedded negative stiffness metamaterial. *J. Intell. Material Syst. Struct.* (2020).

2. Dwivedi, A., Banerjee, A., Adhikari, S. & Bhattacharya, B. Optimal electromechanical bandgaps in piezo-embedded mechanical metamaterials. *Int. J. Mech. Mater. Des.* **17**, 419–439 (2021).

3. Dwivedi, A., Banerjee, A., Adhikari, S. & Bhattacharya, B. Bandgap merging with double-negative metabeam. *Mech. Res. Commun.* **122**, 103889 (2022).

4. Majkut, L. Free and forced vibrations of timoshenko beams described by single difference equation. *J. Theor. Appl. Mech.* **47**, 193–210 (2009).

5. Timoshenko, S. On the differential equation for the flexural vibrations of prismatical rods. *Glas. Hrvat. Prirodosl. Drus., Zagreb.* **32**, 55–57 (1920).

6. Mei, C. & Mace, B. Wave reflection and transmission in timoshenko beams and wave analysis of timoshenko beam structures. *J. Vib. Acoust.* **127**, 382–394 (2005).

7. Elishakoff, I., Kaplunov, J. & Nolde, E. Celebrating the centenary of timoshenko's study of effects of shear deformation and rotary inertia. *Appl. Mech. Rev.* **67** (2015).

8. Dwivedi, A., Banerjee, A. & Bhattacharya, B. Dynamics of piezo-embedded negative stiffness mechanical metamaterials: A study on electromechanical bandgaps. In *ASME International Mechanical Engineering Congress and Exposition*, vol. 84478, V001T01A015 (American Society of Mechanical Engineers, 2020).

9. Huang, H., Sun, C. & Huang, G. On the negative effective mass density in acoustic metamaterials. *Int. J. Eng. Sci.* **47**, 610–617 (2009).

10. Dwivedi, A., Banerjee, A. & Bhattacharya, B. A novel approach for maximization of attenuation bandwidth of the piezo-embedded negative stiffness metamaterial. In *Active and passive smart structures and integrated systems XIV*, vol. 11376, 478–485 (SPIE, 2020).
